# Design, Synthesis, and Characterization of New δ Opioid Receptor-Selective Fluorescent Probes and Applications in Single-Molecule Microscopy of Wild-Type Receptors

**DOI:** 10.1021/acs.jmedchem.4c00627

**Published:** 2024-07-24

**Authors:** Antonios Drakopoulos, Zsombor Koszegi, Kerstin Seier, Harald Hübner, Damien Maurel, Rémy Sounier, Sébastien Granier, Peter Gmeiner, Davide Calebiro, Michael Decker

**Affiliations:** † Pharmazeutische und Medizinische Chemie, Institut für Pharmazie und Lebensmittelchemie, Julius-Maximilians-Universität (JMU) Würzburg, Am Hubland, 97074 Würzburg, Germany; ‡ Institute of Metabolism and Systems Research, University of Birmingham, B15 2TT Birmingham, U.K.; § Centre of Membrane Proteins and Receptors, Universities of Birmingham and Nottingham, B15 2TT Birmingham, U.K.; ∥ Institute of Pharmacology and Toxicology, Julius-Maximilians University of Würzburg, Versbacher Strasse 9, 97078 Würzburg, Germany; ⊥ Chair of Pharmaceutical Chemistry, Department of Chemistry and Pharmacy, Friedrich-Alexander University of Erlangen-Nürnberg, 91058 Erlangen, Germany; # Institut de Génomique Fonctionnelle, CNRS, INSERM, Université de Montpellier, 34094 Cedex 5 Montpellier, France

## Abstract

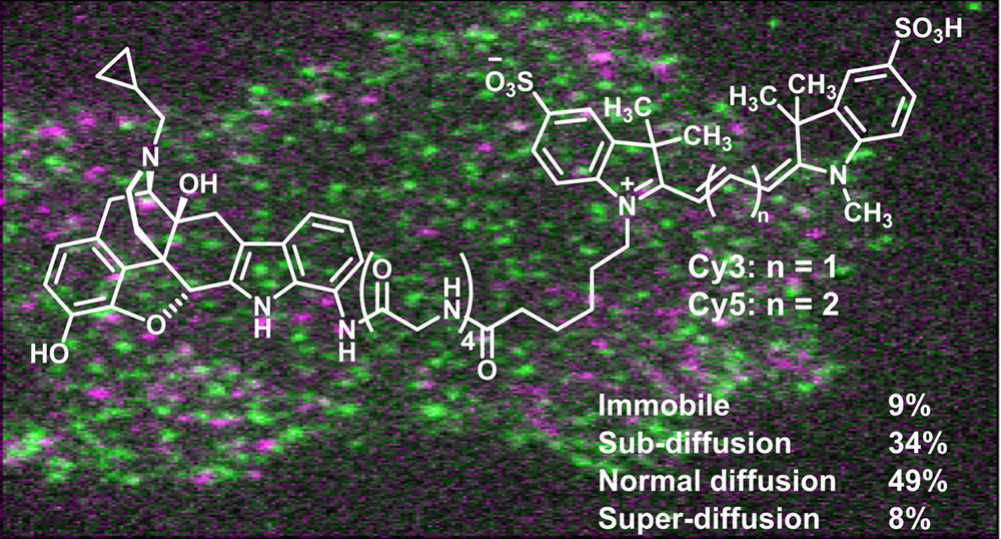

The delta opioid receptor (δOR or DOR) is a G protein-coupled receptor (GPCR) showing a promising profile as a drug target for nociception and analgesia. Herein, we design and synthesize new fluorescent antagonist probes with high δOR selectivity that are ideally suited for single-molecule microscopy (SMM) applications in unmodified, untagged receptors. Using our new probes, we investigated wild-type δOR localization and mobility at low physiological receptor densities for the first time. Furthermore, we investigate the potential formation of δOR homodimers, as such a receptor organization might exhibit distinct pharmacological activity, potentially paving the way for innovative pharmacological therapies. Our findings indicate that the majority of δORs labeled with these probes exist as freely diffusing monomers on the cell surface in a simple cell model. This discovery advances our understanding of OR behavior and offers potential implications for future therapeutic research.

## Introduction

Opium, derived from the plant *Papaver somniferumL.Papaveraceae*, has been used for the management of pain - and recreational purposes - since antiquity.^1,2^ On the one hand, the active ingredients of opium, which belong to the class of phenanthrene alkaloids, cause analgesia, sedation, and euphoria. On the other hand, they are responsible for adverse effects such as respiratory depression, addiction, physical dependence, and mortality.^3−6^ Opioids are currently among the most commonly prescribed pain relievers.^7,8^ The targets of opioid drugs are G protein-coupled receptors (GPCRs) located on neural cell membranes. There are four opioid receptors (ORs):^9^ μ, δ, κ, and the nonclassical nociception receptor (NOR), the structures of which have been elucidated through crystallography and cryoEM.^10−14^

In recent years, the δOR (or DOR) has attracted incromising drug target due to its distinct pharmacological profile and the potential lack of μOR-induced side effects. δORs are primarily expressed in specific regions of the central nervous system (CNS), e.g., olfactory bulb, cortex, striatum, amygdala, hippocampus, hypothalamus, dorsal root ganglia, trigeminal ganglia, and spinal cord, including areas involved in primary pain processing as well as areas responsible for emotional and cognitive aspects of pain.^15−18^ As a result, δOR agonists exhibit antidepressant and anxiolytic effects. Pain management via δOR agonism is particularly effective against chronic pain and migraine with the advantage of low abuse liability and lack of physical dependence. In contrast, it is less effective than μOR-mediated agonism for acute conditions.^19,20^ Furthermore, δOR agonism has been associated with inducing seizures, thus reducing the targets’ attractiveness for pain management. Nonetheless, δOR agonists free of proconvulsive behavior have been reported in the literature, albeit the precise mechanism of δOR agonist-induced seizures remains elusive.^21,22^

Despite the long-held view that, with the exception of family C receptors, most GPCRs are functional as single monomeric units, a number of GPCRs in other families have been proposed to form dimers or even higher-order oligomers.^23^ These include previous studies documenting the formation of μOR (or MOR) and δOR homodimers as well as higher-order homo-oligomers using biochemical methods on membrane preparations.^24^ Moreover, Pascal and Milligan investigated the mechanisms involved in the homodimerization of all ORs via biochemical studies with mutant constructs.^25^ In addition to biochemical methods, emerging fluorescent microscopy techniques during the same period (early 2000s) were used to investigate receptor dimerization. McVey et al. and Ramsay et al. employed Förster resonance energy transfer (FRET), bioluminescence resonance energy transfer (BRET), confocal microscopy as well as Western blot, immunoprecipitation, and ligand binding assays to show κOR (or KOR) and δOR homodimerization.^26,27^ Gomes et al. applied BRET assays to show that μOR and δOR form homodimers.^28^ In 2005, Wang et al. used BRET, immunoprecipitation, and receptor binding assays, to show that all ORs form homodimers and that the formation of dimers occurs before trafficking to the plasma membrane.^29^ Johnston et al. applied computational techniques in combination with BRET, flow cytometry, and binding studies to investigate the prospective modes of δOR homodimerization and suggested that the dimers should have a short lifetime.^30^ While these studies provided new important insights into the existence and mechanisms of OR dimerization, both biochemical and RET approaches typically require overexpression, which may induce dimer formation. Moreover, ensemble approaches do not capture the complex and fast dynamics of protein–protein interactions on the plasma membrane of intact cells, which typically requires the use of single-molecule approaches.^31^

Recent advances in single-molecule microscopy (SMM) techniques have paved the way for their application to study GPCRs at low/physiological expression levels, leading to important findings on receptor localization, organization, and trafficking.^32−39^ Developing fluorescent probes that are suitable for cutting-edge microscopy methods is a valuable asset for both in vitro assays and in vivo imaging.^40−45^ The development of δOR-selective fluorescent probes has made significant progress,^46^ both utilizing a peptide-based,^47−54^ and a morphinane-based design.^55,56^ Moreover, our group has developed highly potent, selective fluorescent probes for the μOR and κOR, which we successfully employed for SMM experiments on living cells.^34−36^ Specifically, we investigated the diffusion profiles of wild-type, untagged ORs on the cell membrane as well as the potential transient homodimer formation. An important innovation of this approach is that it does not require receptor modification, thus circumventing a potential concern that receptor tagging can influence receptor properties, including function, diffusion, dimerization, etc.

### Aim of Project

In the current study, we present the synthesis and pharmacological evaluation of two fluorescent probes with high δOR potency and selectivity based on the antagonist naltrindole (NTI). Furthermore, we used them for SMM experiments to study unmodified δOR localization and diffusion kinetics as well as receptor homodimerization at low/physiological densities.

NTI is an extensively studied, selective δOR antagonist of subnanomolar affinity and is often used as a reference in pharmacological assays.^57−59^ NTI represents a protype application of the “message-address” concept in opioid drug design^60^ and contributed to the experimental verification of the concept when used for the crystallization of δOR.^11^

NTI has been successfully used for the development of bivalent ligands,^61−67^ radiotracers for in vivo imaging,^68−73^ and also fluorescent ligands.^46,55,56^ These studies demonstrate that the optimal positions for implementing a residue or linker without losing potency and selectivity are either on the indole *N* or on carbon *C*-7′. Regarding the chemistry and physicochemical properties of the linker, structure–activity relationships (SARs) from the aforementioned studies suggest that retaining a low overall lipophilicity while achieving an optimal distance between the fluorophore and the binding site is an important factor for δOR selectivity. The incorporation of four hydrophilic glycine groups in the linker has been proven to satisfy this lipophilicity criterion for morphinane analogs, as we have demonstrated in our μOR probe design. There, the first generation of compounds bearing an aliphatic (pentylene) linker had low solubility and produced high background noise, probably due to sticking in the plasma membrane; these drawbacks were amended in the second generation of probes by retaining the pentylene moiety and adding a tetraglycine group. Moreover, the second generation of probes exhibited a higher affinity and selectivity for μOR, presumably due to the longer distance between the two parts of the molecule which should result in fewer interactions between the fluorophore and the binding site.^34,35^ Based on the above, we set out to develop δOR probes that would be suitable for cutting-edge microscopy methods. Cyanine 5 (Cy5) and cyanine 3 (Cy3) are established fluorophores and are used in many state-of-the-art assays.^74−76^ There have been recent reports indicating that the photophysics of cyanine dyes is more complex than previously thought, including lower photostability for dyes bearing an increased polymethine chain, photooxidation leading to sensitivity in oxygen, and FRET fluctuations due to cyanine acceptor blinking.^77−79^ Nonetheless, the Cy5 and Cy3 dyes have been successfully used in many newly developed fluorescent probes for the ORs, while they can also be employed as a FRET pair in relevant studies.^34−36,46,47,51,80,81^

### Chemistry

**Scheme 1:**
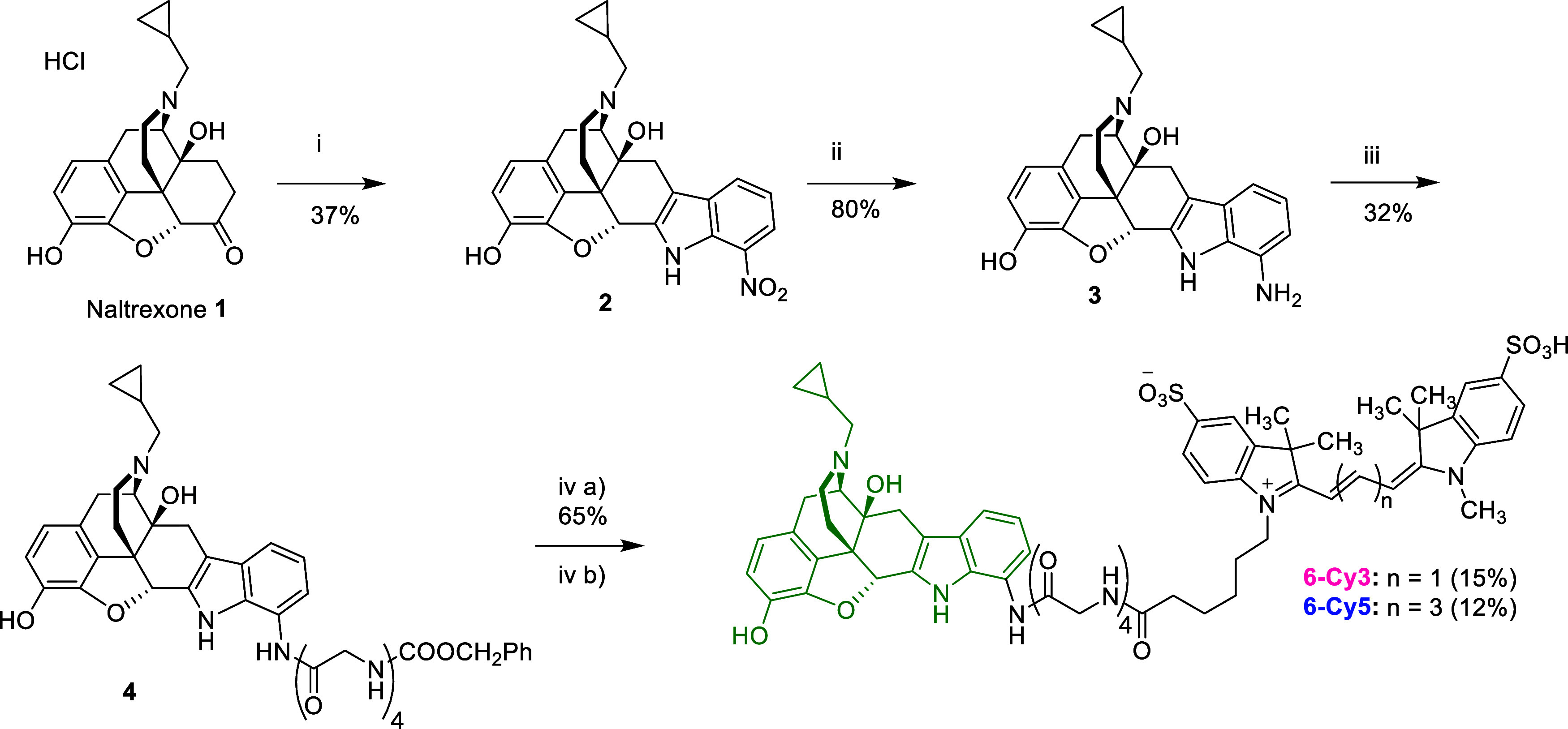
Synthesis of 7′-NTI Coupled to Fluorescent Dyes Cy5 (**6-Cy5**) and Cy3 (**6-Cy3**) via a Tetraglycine Linker Reaction conditions: (i) 2-nitrophenylhydrazine HCl salt/conc. HCl/glacial AcOH ; (ii) Raney–Ni/H_2_NNH_2_/EtOH; (iii) *N*-Cbz-Gly_4_/EDCI/HOBt/Py; (iv) a) TFA 90%/DCM; (b) Cy3-NHS/DIPEA/DMF for **6-Cy3** and Cy5-NHS/DIPEA/DMF for **6-Cy5**.

Inspired by the aforementioned NTI-based studies and previous investigations of our group,^34−36^ we designed a pair of fluorescent ligands bearing a 19-bond linker containing a tetraglycine moiety to connect NTI with the fluorescent dyes Cy3 and Cy5. 7′-Nitro-NTI **2** was synthesized from naltrexone via Fischer indole synthesis (Scheme 1). The reaction yields were relatively low, with the highest yield being 37%. This was due to the unfavorable *ortho*- position of the nitro-group on the phenylhydrazine. The electron-withdrawing effect of the nitro-group led to a deactivation of the hydrazine group, while their close proximity possibly results in a noncovalent intramolecular stabilization system of the respective mesomeric form. Thus, the nucleophilicity of the hydrazine group was reduced, resulting in low yields. The nitro- group of compound **2** was reduced to an amine, yielding 7′-amino-NTI **3** (Scheme 1). The reduction was conducted using Raney nickel catalyst and hydrazine as a hydrogen source. A reduction protocol with Pd/C and hydrogen gas (50 psi) in an acidic buffer was also trialed. It is worth mentioning that the presence of an HCl buffer in this protocol resulted in a monochlorinated byproduct of compound **3**, probably on aromatic position C-1 or C-2.^82^ To avoid the formation of this byproduct, the strongly acidic buffer was switched to an acetic acid system. Both methods provided good yields. However, reduction with Raney nickel was faster, cheaper, easier to filter off, and to work up, and was thus selected as the method of choice.^83^ Compound **3** was then coupled to *N*-Cbz-protected tetraglycine, yielding compound **4** and, after Cbz deprotection via TFA, precursor **5**, which was coupled to Cy3/5-NHS ester and purified via prep HPLC (Scheme 1).^56,66,84^ Coupling compound **3** to *N*-Cbz-Gly_4_ proved to be the bottleneck of the synthetic approach, primarily since the aniline in the 7′ position of the indole moiety is a very poor nucleophile and also due to the low solubility of the polar *N*-Cbz-Gly_4_ in DMF; in order to amend the latter, the medium was switched to pyridine.^85^ Since cleavage of the Cbz protection group was extensively trialed with standard catalytic hydrogenation protocols but with no effect, we opted for acidic deprotection protocols. The anhydrous TFA method was selected since no peptidic bond cleavage from the tetraglycine moiety was observed.

### Pharmacology

#### Affinity Selectivity

**Figure 1:**
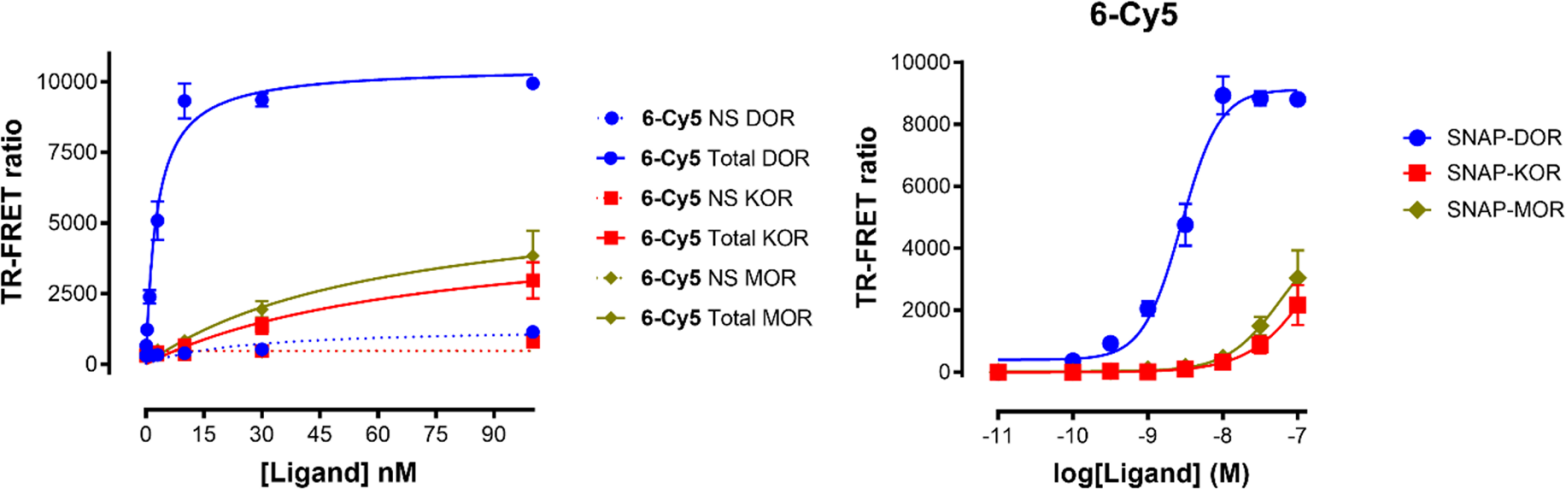
HEK293Τ cells transfected with SNAP-opioid receptors were labeled with Lumi4-Tb and incubated for 1 h with increasing concentrations of **6-Cy5** at room temperature. Data are mean ± SD of *n* = 3 independent experiments. Left: Total and nonspecific binding. Right: Right: Data (specific binding) presented in semilogarithmic dose-response sigmoidal graph.

The affinity and selectivity profile of compound **6-Cy5** was determined by a fluorescence assay based on homogeneous time-resolved FRET (HTRF). SNAP-opioid receptors expressed on the surface of HEK293T cells were labeled with nonpermeant SNAP-tag substrates derivatized with the dye Lumi4-Tb (SNAP-Lumi4-Tb) which acts as a FRET donor. Upon binding of fluorescent ligands acting as FRET acceptors on SNAP-opioid receptors, the HTRF signal from the sensitized acceptor can be detected. Based on this signal, a saturation curve was plotted by increasing the concentrations of the examined fluorescent compound, allowing the determination of binding affinity. Nonspecific binding was determined by the addition of a molar excess of naloxone (100 μM). Thus, only **6-Cy5** was measured using this assay (Figure 1), because Cy3 is not a FRET acceptor in a pair with Lumi4-Tb. The estimated *K*_d_ values were 1.8 ± 0.8 nM (δOR), 215 ± 262 nM (μOR), and 226 ± 131 nM (κOR), proving high affinity for δOR. Nonetheless, the *K*_d_ values for μOR and κOR exhibit a high uncertainty in the measurement, which originates from the fact that the compound is not binding as well with μOR and κOR as with δOR, and a ligand saturation level cannot be reached for these receptors within the concentration range of the specific assay setting (Figure 1).

In addition, concentration-binding curves were conducted using TIRF microscopy of cells overexpressing human δOR after overnight transient transfection. In order to estimate the binding affinity of both compounds (**6-Cy3** and **6-Cy5**), Chinese hamster ovary (CHO) cells transiently expressing human δOR were incubated with various concentrations of the ligands. Images of ligand-bound cell surface receptors were taken on a TIRF microscope, whereby the total internal reflection methodology excites only the fluorophores located within 100–200 nm of the interface between the glass coverslip and the cells adhering to it. The average intensity of labeled cells was compared and fitted with a logarithmic response curve giving a *K*_d_ of 2.3 ± 0.9 nM for compound **6-Cy3** and 5.7 ± 2.4 nM for compound **6-Cy5** (Figure S4).

Furthermore, radioligand binding studies were conducted for compounds **6-Cy3** and **6-Cy5** using membrane preparations from HEK293T cells. Receptor densities (*B*_max_ value) and specific binding affinities (*K*_d_ value) for the radioligand [^3^H]diprenorphine (specific activity 31 Ci/mmol) were estimated to be 1,900 fmol/mg protein, 0.27 nM for δOR, 4,400 fmol/mg protein, 0.12 nM for κOR, and 1900 fmol/mg protein, 0.090 nM for μOR, respectively. Nonspecific binding was determined in the presence of naloxone at a final concentration of 10 μM. The resulting K_i_ values showed OR affinities of *K*_i_ = 1.7 nM (δOR); *K*_i_ = 370 nM (μOR); *K*_i_ = 330 nM (κOR) for **6-Cy3**, and *K*_i_ values = 1.2 nM (δOR); *K*_i_ = 100 nM (μOR); *K*_i_ = 78 nM (κOR) for **6-Cy5**, respectively. The resulting *K*_d_ and *K*_i_ values from all methods are in good agreement with each other (Table S4).

### Intrinsic Activity

Intrinsic activity measurements of compounds **6-Cy3 and 6-Cy5** were conducted to verify that they both retained the pharmacological profile of the parent compound, naltrindole. Efficacy for G protein activation was measured via the IP-One HTRF assay (Cisbio, Codolet, France), under cotransfection of δOR and the hybrid G protein Gα_qi_, a Gα_q_ protein with the last five C-terminal amino acids replaced by the corresponding sequence of Gα_i_. Furthermore, β-arrestin-2 recruitment was measured using the PathHunter assay (DiscoverX, Birmingham, U.K.). In both assays, leu-enkephalin was used as a reference δOR agonist in transiently transfected HEK293T cell preparations for the IP-One assay and transiently transfected HEK293T cells stably expressing the enzyme acceptor tagged β-arrestin-2 fusion protein.^86−88^ It was shown that both compounds **6-Cy3** and **6-Cy5** did not elicit a response when studied in agonist mode. Antagonist properties for the ligands were determined when **6-Cy3** inhibited an EC_80_ concentration of leu-enkephalin with an IC_50_ of 38 nM in the IP_1_ assay and 47 nM in the arrestin recruitment assay. Similar properties were measured for **6-Cy5** with IC_50_ values of 54 nM (IP_1_) and 33 nM (arrestin) (Figures S1 and S2 and Table S3). These measurements, combined with the well-studied pharmacology of the parent ligand naltrindole, indicate that the fluorescent probes share the same antagonistic profile as naltrindole.

### Dissociation Kinetics

**Figure 2:**
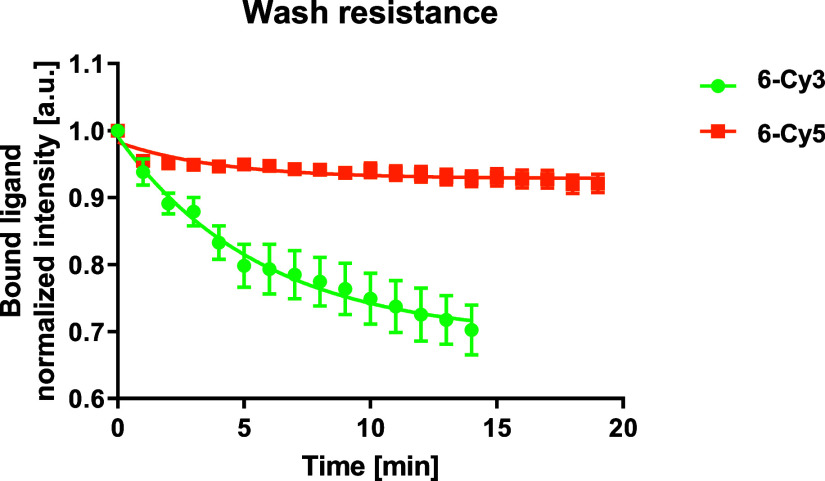
Normalized fluorescence intensity plotted over time and fitted with a one phase exponential decay function for fluorescent probes **6-Cy3** and **6-Cy5**. Data are mean ± SEM of *n* = 3 independent experiments.

Fluorescent probes with slow dissociation kinetics are preferable for imaging applications, as this allows washing out unbound probes and retaining a high degree of labeling during imaging. This classifies the wash resistance of the noncovalently bound fluorescent probes as a property of paramount importance: should the probes wash out from their binding site very fast, then the respective receptors will quickly become invisible to an SMM setup. Therefore, dissociation kinetics measurements were performed using TIRF microscopy in overexpressing transiently transfected CHO cells for compounds **6-Cy3** and **6-Cy5**. Compound **6-Cy3** showed a wash resistance of 69 ± 1% (*R*^2^ = 0.99), while compound **6-Cy5** a wash resistance of 93 ± 1% (*R*^2^ = 0.81) after 15 and 20 min of medium wash out, respectively (Figure 2).

### Single-Molecule Microscopy

**Figure 3:**
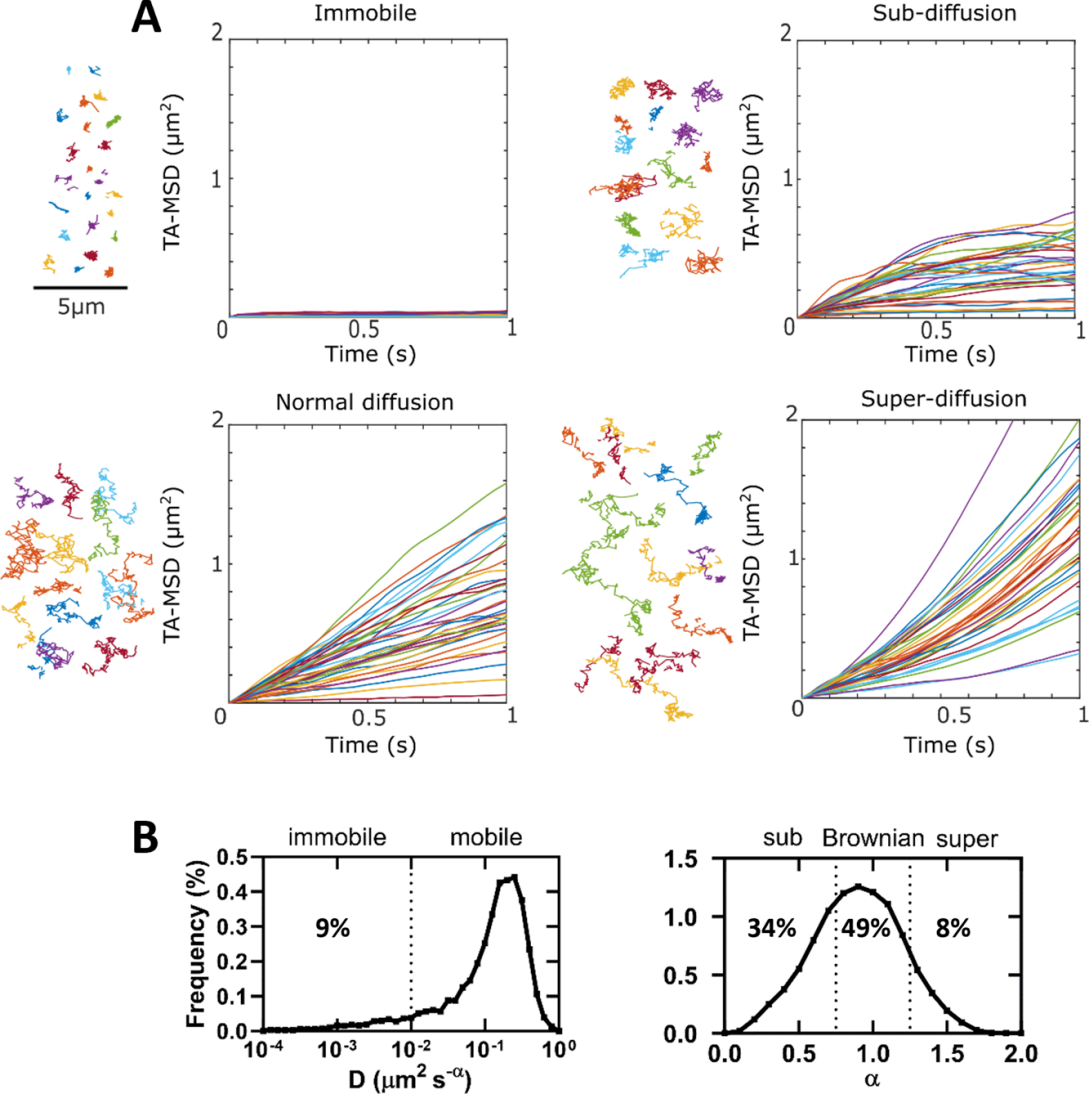
Time-averaged mean squared displacement (TA-MSD) analysis of δOR trajectories using compound **6-Cy3**. (A) Representative trajectories for each type of motion together with the corresponding TA-MSD plots. (B) Distribution of generalized diffusion coefficient (*D*) and the anomalous diffusion exponent (α) values estimated for the analyzed trajectories. Data are from 7774 individual trajectories from 54 movies.

The TIRF microscopy experiments assessing binding affinity and wash resistance had proven that compounds **6-Cy3** and **6-Cy5** exhibit suitable fluorescent properties for SMM. However, Cy5 is more prone to photobleaching in comparison to Cy3.^89^ Furthermore, the labeling specificity of the probes was tested by incubating them with nontransfected cells, which did not show any signal in SMM (Figure S3). To investigate the diffusion behavior of the δOR, CHO cells were transiently transfected with the wild-type human δOR receptor. Four to 6 h after transfection, the cells were labeled with a saturating concentration of compounds **6-Cy3** and **6-Cy5** for 20 min. After a washing step, fast, multicolor single-molecule imaging was performed on a custom four-camera TIRF microscope at a rate of one image every 30 ms. The receptor density was low enough to perform automated single-particle detection and tracking. The Cy5-labeled particles had an average density of 0.22 ± 0.05 receptors/μm^2^ (minimum density: 0.12 receptors/μm^2^, maximum density: 0.32 receptors/μm^2^) and the Cy3-labeled particles had an average density of 0.39 ± 0.07 receptors/μm^2^ (minimum density: 0.24 receptors/μm^2^, maximum density: 0.50 receptors/μm^2^). A time-averaged mean squared displacement (TA-MSD) analysis on the particle trajectories was used to evaluate their diffusion (Figure 3A). Our single-particle tracking experiments are done under relatively mild illumination conditions. Under such conditions, blinking is negligible and photobleaching is kept to a minimum. Photobleaching is measured and corrected for in all our quantitative analyses by incorporating it in the deconvolution when examining interactions. The MSD analysis uses at least 100 frame long trajectories, if a particle photobleaches faster, then it is omitted from further analysis. Since the TA-MSD analysis revealed heterogeneity in diffusional behavior among particles, their trajectories were categorized according to the diffusion parameters *D* (diffusion coefficient) and alpha α (anomalous diffusion exponent). Particles with *D* < 0.01 μm^2^s^–α^ were considered to be immobile. Normal diffusion was assigned to particles that had *D* ≥ 0.01 μm^2^s^–α^ and 0.75 ≤ α ≤ 1.25. Sub- and superdiffusion were assigned to particles with *D* ≥ 0.01 μm^2^s^–α^ and α < 0.75 or α > 1.25, respectively (for more details cf. Experimental Part – Single Molecule Microscopy). This categorization showed that 9% of the receptors were virtually immobile, 34% were characterized by a subdiffusive behavior, 49% were consistent with normal diffusion, and 8% were superdiffusive (Figure 3B).

**Figure 4:**
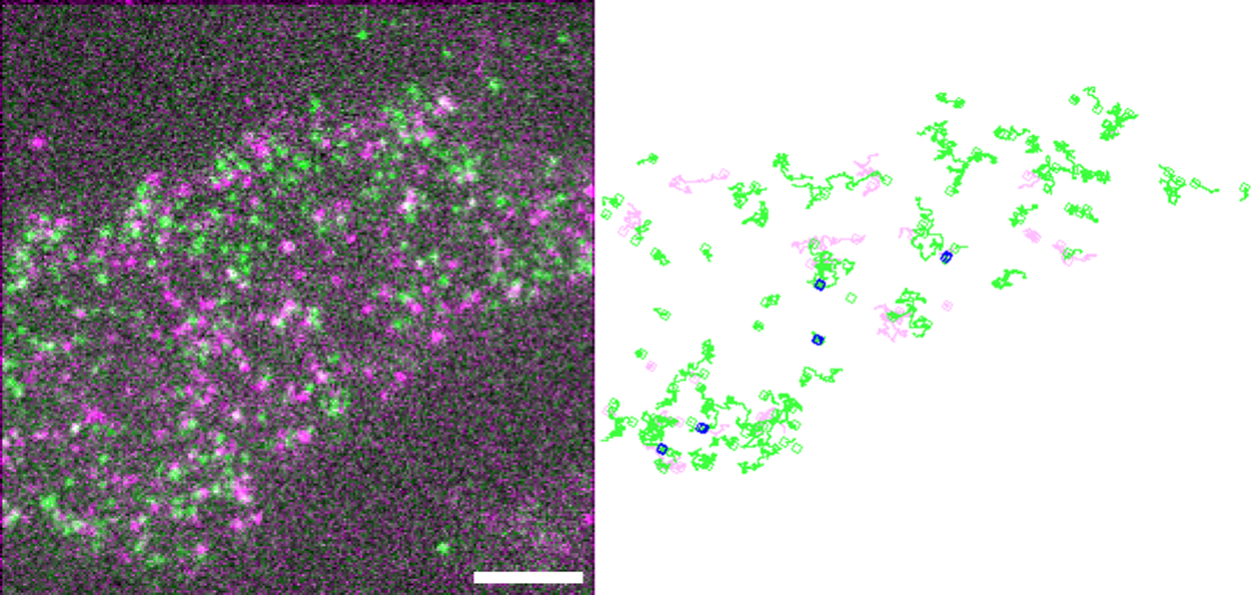
Two-color single-molecule imaging of δORs in CHO cells transiently transfected with human δOR and labeled with 100 nM of compounds **6-Cy3** and **6-Cy5** for 20 min, followed by a washing step. A representative cell is shown (left); scale bar: 5 μm. Representative trajectories are shown (right), green: **6-Cy3**, magenta: **6-Cy5**, blue: colocalizations.

**Figure 5:**
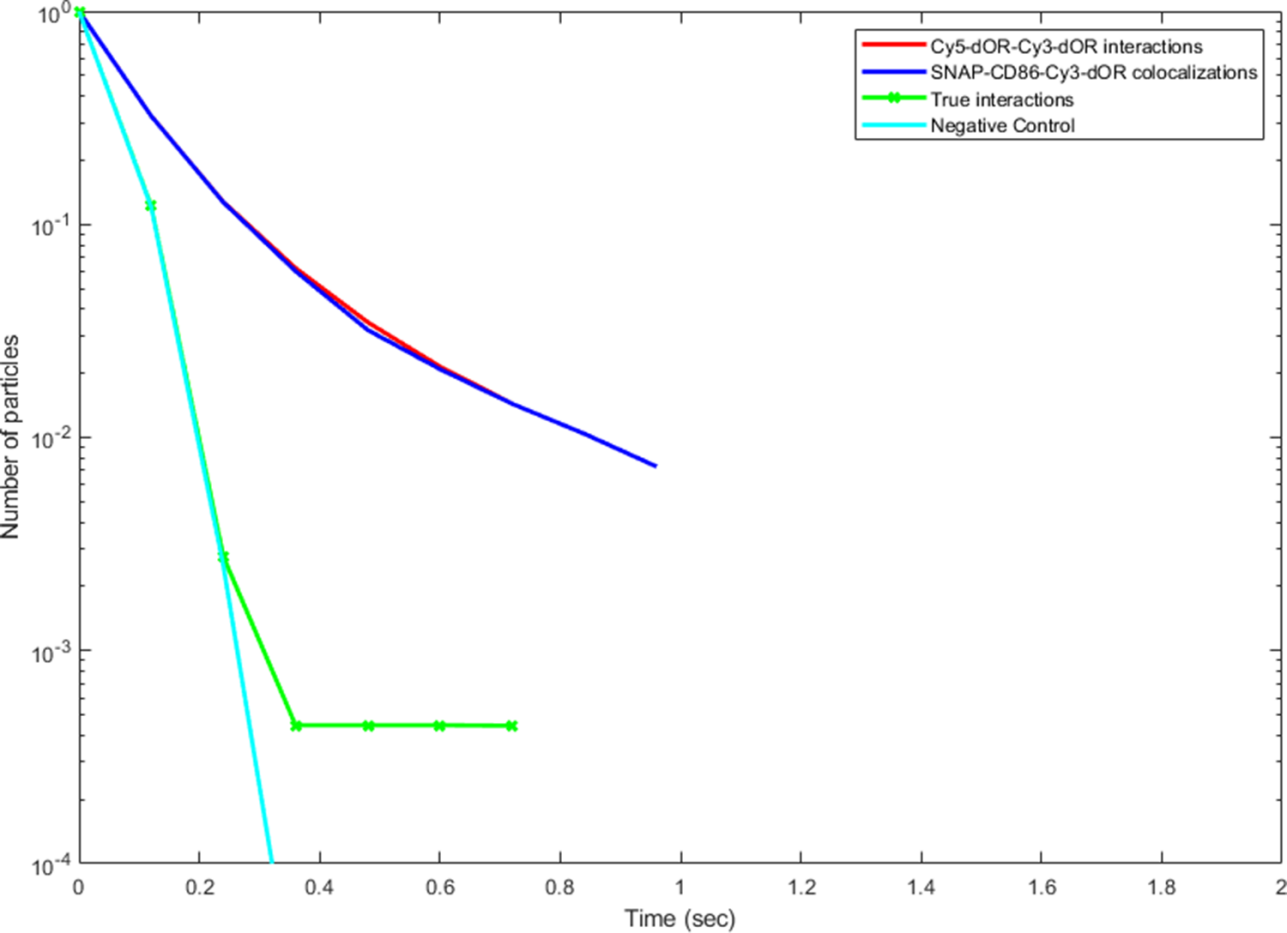
Results of deconvolution-based analysis to estimate δOR transient interactions. *N* = 55,341 and 27,491 interactions from 54 and 24 individual cells for δOR-δOR and δOR-CD86, respectively. The curve corresponding to true interactions is very close to the negative control, indicating the lack of detectable transient interactions.

To investigate whether δOR forms dimers at the aforementioned densities, two-color single-molecule experiments were performed, using δORs labeled with compounds **6-Cy3** and **6-Cy5** simultaneously (Figure 4). In order to estimate the true dimer lifetime, a control (SNAP-CD86) was introduced. CD86 is a known unrelated transmembrane protein that does not interact with ORs and was labeled with an Alexa647-conjugated SNAP substrate. First, the distribution of colocalization times between δOR-**6-Cy3** and δOR-**6-Cy5** was measured. Then, by measuring the colocalization times between δOR-**6-Cy3** and SNAP-CD86 molecules, we were able to determine the distribution of random colocalizations. After deconvolution of the δOR-**6-Cy3** and δOR-**6-Cy5** colocalizations with the ones obtained for δOR-**6-Cy3** and SNAP-CD86, the true receptor-receptor dimer lifetime can be estimated.^90−92^ The results showed no significant difference between δOR-**6-Cy3** and δOR-**6-Cy5**, and between δORs-**6-Cy3** and SNAP-CD86 colocalization times, consistent with the lack of detectable transient dimerization events (Figure 5).

## Discussion

Affinity of **6-Cy5** and **6-Cy3** is in the same range as NTI4F,^56^ with δOR selectivity being at the same level or better. These results classify the compounds among the best nonpeptidic δOR-selective fluorescent probes reported in terms of potency, selectivity, and wash resistance. Furthermore, they retain the pharmacological profile of the parent ligand NTI for both G protein activation and β-arrestin-2 recruitment. Their excellent optical properties and slow dissociation kinetics render them particularly suited for advanced microscopy approaches, spanning from SMM to in vivo imaging of endogenous receptors. In cells transfected with wild-type δOR labeled with the **6-Cy3** ligand, individual **6-Cy3** labeled δOR molecules could be observed diffusing on the plasma membrane even after 2–3 h following labeling. By applying one-color SMM, we were able to investigate the diffusion behavior of wild-type δOR under low/physiological expression levels in living cells. The analysis revealed a heterogeneous distribution for the different types of motion with a small immobile fraction of 9%. This is lower than the observations made by our group using the same methodology for other opioid receptors (Table 1).^34−36^ Receptors showing a subdiffusive behavior were 34%, similar to the μOR (Table 1). The biggest fraction of the receptors (49%) exhibited normal diffusion (i.e., Brownian motion), like what we had observed for κOR (Table 1). Approximately 8% of δOR were superdiffusive, with the values for all three ORs being very similar (Table 1). The differences among the ORs can be explained by differences among the receptors as well as by the different nature of the ligands used. It has been shown in previous studies that such a complicated diffusion behavior is not unique to GPCRs, but it is characteristic of other membrane proteins also. This diffusion profile can be attributed to a series of factors that interact with each other: trapping of receptors in small membrane compartments, receptor-G protein interactions occurring at “hot spots”, i.e., low potential energy areas, important roles of the cytoskeleton, lipid, and actin fiber nanodomains as well as clathrin-coated pits. Overall, a better understanding of the complex dynamics at the basis of GPCR-protein interactions can lead to the development of innovative drugs that can module GPCR signaling in a sophisticated way.^37,91,93^

**Table 1:** Diffusion Profiles of Human δOR, μOR and κOR Studied by SMM via Fluorescent Ligand Labeling

	δOR (%)	μOR (%)^34,35^	κΟR (%)^36^
immobile	9	22	16
subdiffusion	34	34	25
normal diffusion	49	34	51
superdiffusion	8	10	8

Studies have shown that the efficacy of a ligand in general (i.e., agonism, antagonism, inverse agonism, biased agonism) influences GPCR diffusion behavior and affects potential dimerization. More specifically, in SNAP-tagged μOR, the biased agonist DAMGO caused a transient increase in the diffusion speed of the receptors and enhanced dimer formation, while morphine did not.^37^ Also, SMM investigations of SNAP-tagged and wild-type dopamine receptors labeled with fluorescent probes, led to a significant increase in dimerization and mobility when the receptors were bound by agonists but not byantagonists.^94^ Hence, it would be interesting to synthesize in the future fluorescent ligands with a variety of pharmacological profiles (e.g., partial agonists, full agonists, inverse agonists, biased agonism toward β-arrestin recruitment, etc.) to investigate how they alternate the diffusion and dimerization patterns of each wild type OR. The data presented in Table 1 can exemplarily showcase this: the δOR fluorescent probes were purely antagonistic, while for κOR the antagonist fluorescent probes slightly promoted the recruitment of β-arrestin. In the case of the μOR, the fluorescent ligands were based on a partial agonist resulting in slightly slower diffusion of the monomers and homodimer detection. Those differences in ligands could explain the differences in diffusion behavior among ORs.

A recent study on OR homodimerization employing advanced microscopy methods was conducted by Cechova et al. in 2021.^38^ This study employed GFP and SNAP-tag labeled ORs expressed at densities of 5, 10–100, and 150 receptors/μm^2^ to detect homodimers on the surface of living cells in the absence of OR ligands. No δOR homodimers were found at the aforementioned densities.^38^ However, there have been reports of lower OR densities found on the plasma membrane of physiological neurons in the CNS (<1 receptors/μm^2^),^95,96^ which, to our knowledge, have not been addressed either for their diffusion behavior or the prospective homodimerization of δOR, until now. We conducted dual-color SMM measurements to investigate δOR homodimerization in a density range of 0.1–0.5 receptors/μm^2^ on the plasma membrane. Our measurements with untagged receptors did not detect a fraction of long-lasting (>100 ms) δOR dimers on the surface of living cells (Figure 5).

Our results are in agreement with the observations of Cechova et al. for higher δOR receptor densities, albeit without the presence of a ligand, as the measurements of that study were conducted using unliganded tagged receptors.^38^ This further supports the quality of our method, highlighting its potential application for visualizing endogenous, unmodified receptors; which is a limitation of methods based on tagged/engineered receptors.

As analyzed above for the diffusion behavior, a possible underlying relation of the ligand profiling with the receptors’ dimerization promotion may be inferred also for receptor dimerization. The purely antagonistic probes in the current investigation, as well as the antagonistic but low β-arrestin recruiting probes in κOR, when bound to the receptors, did not trigger homodimerization, while the partially agonistic probes (but not β-arrestin recruiting) in μOR did exhibit a low percentage of homodimer organization.^34−36^ As we have previously remarked, stabilization of different receptor conformations as a result of ligand binding may promote or eliminate dimerization and thus result in different pharmacological responses.^67^ Therefore, exploring the influence of various different pharmacologic profiles of prospective future probes would be worthwhile also for investigating receptor dimerization, in addition to diffusion patterns, as mentioned above, with the perspective of applying them to endogenous receptors.

## Conclusions

A pair of δOR-selective fluorescent probes was designed, synthesized, and characterized. Compounds **6-Cy3** and **6-Cy5** exhibit excellent optical properties and pharmacological profiles, which ranks them among the best nonpeptidic δOR-selective fluorescent probes reported. The compounds were successfully applied in SMM and revealed heterogeneous diffusion behavior for wild-type δOR. A smaller fraction of virtually immobile receptors was observed in comparison to the μOR and κOR using the same technique. This may be explained by the different pharmacologic properties of the fluorescent ligands employed in each study. Furthermore, they can prove to be valuable in labeling endogenous ORs in natural tissue.

## Experimental Part

### Chemistry Part

#### Materials and Methods

##### Chemicals

The fluorescent dyes Cy3 NHS ester and Cy5 NHS Na^+^ ester were purchased from Click Chemistry Tools. Starting material naltrexone was purchased from Carbosynth. All other chemicals and solvents were purchased from Sigma-Aldrich.

##### Thin-Layer Chromatography

Thin-layer chromatography for reaction control purposes was conducted on coated plates (Silica Gel 60 F254). The visualization of the substances was performed by the following methods: (a) fluorescence, when irradiated with UV-light (256 nm), (b) spray-reagents (Dragendorff’s reagent, Ehrlich’s reagent), and (c) coloring in iodine chamber.

##### Column-Chromatography

Manual column chromatography was performed using silica gel with a grain size of 0.063–0.2 mm (company Merck, Darmstadt, Germany) and wet packing. The composition of the eluent systems is indicated in percentages by volume.

##### Nuclear Magnetic Resonance Spectroscopy

NMR spectra were recorded on a Bruker AV 400 FT-NMR-spectrometer (company Bruker Biospin, Karlsruhe, Germany) (^1^H: 400 MHz) at room temperature. The residual protons and the resonance signals of the deuterated solvents were used as an internal standard. The chemical shifts δ were reported in parts per million, and the coupling constants were reported in [Hz]. The signal multiplicities follow the abbreviations: s = singlet, d = doublet, t = triplet, q = quartet, dd = doublet of doublets, and m = multiplet.

##### Liquid Chromatography–Mass Spectrometry Data

The analytical HPLC was conducted on a Shimadzu LC20AB system equipped with a DGU-20A3R controller and an SPD-20A UV/vis detector, while a Synergi 4u Fusion-RP (150 × 4.6 mm) column served as the stationary phase. Gradient MeOH + 0.1% formic acid (phase A) and water +0.1% formic acid (phase B) were used as mobile phases (cf. Tables S5–S8). The analytical HPLC flow rate was 1 mL/min. ESI-MS spectral data were acquired on a Shimadzu LCMS-2020 single quadrupole LC-MS instrument (Shimadzu Europe, Duisburg, Germany). High mass accuracy and resolution experiments were performed on a Bruker Daltonics timsTOF Pro spectrometer by using electrospray ionization (ESI) as an ionization source. The purification of the precursor **5** and target compounds **6-Cy3** and **6-Cy5** was performed via semipreparative HPLC on the above Shimadzu system using a puri a Synergi 4u Fusion-RP 80A (250 × 10.0 mm) column as stationary phase and flow rates of 2.5–3 mL/min. All compounds had a purity >95% in LC-MS. The gradient systems used are described in the SI (cf. Tables S1and S2).

### Syntheses

The IUPAC names of the target compounds **6-Cy3**, and **6-Cy5** were generated using ChemDraw 20.1.1. The fluorescent dyes were reacted as zwitterions. However, due to the 0.1% formic acid in the LCMS gradient system, protonation occurs, leading to a +1 of the calculated mass M. Noted with #. The low amount of the final target compounds **6-Cy3 and 6-Cy5** did not allow measurement of NMR spectra, but LCMS and HRMS spectra proved both compound identity and purity.

### 7′-Nitronaltrindole (2)

Naltrexone HCl salt (377 mg,1 equiv) and 2-nitrophenylhydrazine HCl salt (189 mg, 1 equiv) were dissolved in a concentrated HCl: glacial acetic acid 1:1 v/v mixture. The reactants were fully dissolved once the temperature started rising. The reaction mixture was heated at 86 °C for 6 h and 90 °C for 12 h under a nitrogen atmosphere and stirring. TLC and LCMS control suggested that the reaction had concluded. After cooling, the reaction mixture was basified with solid Na_2_CO_3_ until saturation, resulting in a semisolid mixture. Brine was added to make the mixture more soluble, and it was extracted with an excessive amount of dichloromethane and chloroform. The combined organic extract was distilled to reduce its volume and washed with brine. The organic phase was then dried (Na_2_SO_4_) and evaporated under reduced pressure to yield a dark solid residue. Purification through column chromatography (ethyl acetate:petroleum ether: NH_3_ – 1:1:0.01) yielded 170 mg of yellow solid. Yield 37%.^1^H NMR (CDCl_3_, 400 MHz) δ [ppm]: 9.97 (s, 1H, NH), 8.07 (m, 1H, H6′), 7.75 (d, *J* = 7.7 Hz, 1H, H4′), 7.10 (t, *J* = 7.9 Hz, 1H,H5′), 6.70 (d, 1H, *J* = 8.1 Hz, H2), 6.60 (d, *J* = 8.1 Hz, 1H, H1), 5.71 (s, 1H, H5), 3.40 (d, *J* = 6.5 Hz, 1H, H9), 3.19 (d, *J* = 18.7 Hz, 1H, H10b), 2.92 (d, *J* = 15.7 Hz, 1H, H15b), 2.80 (m, 2H, H10a and), 2.63 (d, *J* = 15.8 Hz, 1H, H15a), 2.45 (m, 3H, H8b and N–CH_2_-CH-(CH_2_)_2_), 2.32 (m, 1H, H16b), 2.04 (s, 1H, OH), 1.82 (m, 1H, H8a), 1.24 (m, 1H, H16a), 0.89 (m, 1H, N–CH_2_–CH–(CH_2_)_2_), 0.61 (m, 2H, N–CH_2_–CH–(CH_2_)_2_), 0.19 (m, 2H, N–CH_2_–CH-(CH_2_)_2_). ^13^C NMR (101 MHz, CDCl_3_) δ 127.13 (1C, C4**′**), 119.95 (1C, C6′), 119.48 (1C, C1), 118.70 (1C, C5′), 117.25 (1C, C2), 84.76 (1C, C5), 62.13 (1C, C9), 59.47 (1C, N–CH_2_–CH–(CH_2_)_2_), 43.60 (1C, C16), 31.45 (1C, C8), 28.65 (1C, C15), 23.12 (1C, C10), 9.40 (1C, N–CH_2_–CH-(CH_2_)_2_), 4.10 (1C, N–CH_2_–CH–(CH_2_)_2_), 3.76 (1C, N–CH_2_–CH-(CH_2_)_2_). MS: C_26_H_25_N_3_O_5_ calc. 459.18. ESI (*m*/*z*): 460.15 [M + H]^+^.

### 7′-Aminonaltrindole (3)

Compound **2** (690 mg, 1 equiv) was well dissolved in 30 mL of ethanol under stirring. Wet Raney–Ni (2 teaspoons), previ water, was added portionwise. Hydrazine (561 μL, 7.7 equiv) was added dropwise to the reaction mixture which was left to stir vigorously at room temperature under a nitrogen atmosphere (balloon). After the addition of hydrazine, the mixture started bubbling and soon afterward the characteristic yellow color disappeared. TLC control after 1h showed that the reaction had finished. A Celite pad was used for filtering the reaction mixture and the cake was thoroughly washed with ethanol, methanol, and boiling methanol. The volume of the filtrates’ combined organic fractions was reduced under vacuum, dried (Na_2_SO_4_), and filtered, and the crude was taken to dryness and left at the desiccator under vacuum overnight. LCMS check showed excellent purity, with no need for column purification. 516 mg. Yield 80%.^1^H NMR (400 MHz, MeOD) δ [ppm]: 6.82 (m, 2H, H6′ and H5′), 6.62 (s, 2H, H1 and H2), 6.53 (dd, *J* = 7.0, 1.4 Hz, 1H, H4′), 5.66 (s, 1H, H5), 3.86 (d, *J* = 5.9 Hz, 1H, H9), 3.29 (m, 1H, H10b), 3.13 (dd, *J* = 19.3, 6.7 Hz, 1H, H10a), 2.93 (m, 3H, H16b, OH, N–CH_2_-CH-(CH_2_)_2_), 2.76 (dd, *J* = 13.1, 7.0 Hz, 1H, N–CH_2_-CH-(CH_2_)_2_), 2.61 (m, 3H, H16a, H8b, OH), 1.92 (s, 2H, NH_2_), 1.81 (d, *J* = 11.5 Hz, 1H, H15b), 1.30 (m, 2H, H15a, H8a), 1.05 (ddd, *J* = 12.4, 7.5, 4.9 Hz, 1H, N–CH_2_–CH-(CH_2_)_2_), 0.72 (dt, *J* = 18.1, 8.5 Hz, 2H, N–CH_2_–CH-(CH_2_)_2_), 0.39 (m, 2H, N–CH_2_–CH-(CH_2_)_2_). ^13^C NMR (101 MHz, MeOD) δ 121.04 (1C, C5′), 120.19 (1C, C2), 118.98 (1C, C1), 110.33 (1C, C6′), 109.27 (1C, C4′), 85.70 (1C, C5), 63.72 (1C, C9), 59.47 (1C, N–CH_2_–CH–(CH_2_)_2_), 46.57 (1C, C16), 30.90 (1C, C8), 30.30 (1C, C15), 24.66 (1C, C10), 8.13 (1C, N–CH_2_–CH-(CH_2_)_2_), 5.61 (1C, N–CH_2_–CH-(CH_2_)_2_), 3.63 (1C, N–CH_2_–CH-(CH_2_)_2_). MS: C_26_H_27_N_3_O_3_ calc. 429.21. ESI (*m*/*z*): 430.20 [M + H]^+^.

### 7′-(*N*-Cbz-tetraglycyl)-amidonaltrindole (4)

The *N*-Cbz-Gly_4_ (252 mg, 1.1 equiv) was treated with 5 mL of dry pyridine under stirring and Ar, leading to a light suspension. After ice bath cooling, EDCI HCl (127 mg, 1.1 equiv) and HOBt (10 mg, 0.1 equiv) were added and the mixture was left to stir at 0 °C under Ar for 1 h. Then 7′-aminonaltrindole **3** was added (258 mg, 1 equiv). The reaction mixture was left to stir under Ar and gradually come to rt for 3 overnights. TLC and LC–MS control showed that the reaction was concluded and the reaction mixture was taken to dryness. The crude residue was kept overnight in the desiccator and was worked up with ethyl acetate and saturated NaHCO_3_. The combined organic phases were dried (Na_2_SO_4_), filtered, and taken to dryness (354 mg crude product). Purification via column chromatography (dichloromethane: methanol:NH_3_ 20:1:0.1 → 10:1:0.1). 150 mg. Yield: 32%.^1^H NMR (400 MHz, MeOD) δ [ppm]: 8.46 (br. s., 1H, NH), 7.31 (m, 7H, H6′, H4′, H18″-H22”), 6.97 (t, *J* = 7.6 Hz, 1H, H5′), 6.70 (s,2H, H1 and H2), 5.72 (s, 1H, H5), 5.01 (s, 2H, H16”), 4.15 (q, *J* = 17.2 Hz, 3H, H9, H16b, H10b), 3.83 (m, 8H, H3″, H6″, H9″, H12”), 3.26 (br. s, 1H, H10a) 3.08 (br. s, 1H, H8b), 2.95 (d, *J* = 15.8 Hz, 2H, N–CH_2_-CH-(CH_2_)_2_), 2.72 (m, 3H, H8a, H16a, H15b), 1.81 (br. s, 1H, H15a), 1.11 (m, 1H, N–CH_2_–CH-(CH_2_)_2_), 0.80 (d, *J* = 39.9 Hz, 2H, N–CH_2_–CH-(CH_2_)_2_), 0.51 (br. s, 2H, N–CH_2_–CH-(CH_2_)_2_). ^13^C NMR (101 MHz, MeOD) δ 128.06 (2C, C19″, C21”), 127.64 (1C, C20”), 127.45 (2C, C18″, C22”), 119.36 (1C, C1), 118.90 (1C, C5′), 118.03 (1C, C2), 116.96 (1C, C4′), 116.23 (1C, C6′), 83.68 (1C, C5), 66.55 (1C, C16”), 62.28 (1C, C9), 57.53 (1C, N–CH_2_–CH–(CH_2_)_2_), 46.00 (1C, C16), 43.56 (1C, C3”), 42.77 (3C, C6″, C9″, C12”), 28.88 (1C, C8), 28.42 (1C, C15), 23.58 (1C, C10), 5.51 (1C, N–CH_2_–CH-(CH_2_)_2_), 4.77 (1C, N–CH_2_–CH-(CH_2_)_2_), 2.03 (1C, N–CH_2_–CH-(CH_2_)_2_). MS: C_42_H_45_N_7_O_9_ calc. 791.33. ESI (*m*/*z*): 792.35 [M + H]^+^, 396.85 [M + 2H]^2+^.

### 7′-Tetraglycylamidonaltrindole (5)

A solution of **4** (5 mg, *M*_W_ = 791.33) dissolved in 2 mL of a TFA:DCM 9:1 solvent was stirred under an Ar atmosphere in RT. The reaction was concluded after 48 h. The reaction mixture was basified with DIPEA under ice cooling (pH = 7–8) and the solvent was evaporated. Acetonitrile and dichloromethane were added in portions to aid the evaporation. The crude product was dissolved in 10 mL of acetonitrile and a small amount of water, with the help of ultrasound and mild heating, and it was purified via prep RP-HPLC (Table S1). Thirty milligrams of a yellow oily film. Yield: 65%.^1^H NMR (400 MHz, MeOD) δ 7.31 (d, 1H, *J* = 7.6 Hz, H6′), 7.22 (d, 1H, *J* = 7.2 Hz, H4′), 6.99 (t, 1H, *J* = 7.8 Hz, H5′), 6.69 (s, 2H, H1, H2), 5.76 (s, 1H, H5), 4.10 (m, 6H, H3″, H6″, H9”), 3.68 (m, 1H, H16b), 3.56 (m, 1H, H9), 2.94 (m, 2H, N–CH_2_-CH-(CH_2_)_2_), 2.72 (dd, *J* = 24.8, 10.4 Hz, 2H, 8b, 16a), 2.53 (t, *J* = 7.1 Hz, 2H, NH_2_), 2.33 (m, 2H, H8a, H15b), 1.88 (m, 2H, NH, OH), 1.67 (m, 2H, H10b, NH), 1.56 (m, 2H, H10a, NH), 1.33 (m, 2H, H15a, NH), 1.14 (m, 1H, N–CH_2_–CH-(CH_2_)_2_), 0.83 (m, 2H, N–CH_2_–CH-(CH_2_)_2_), 0.54 (t, *J* = 4.7 Hz, 2H, N–CH_2_–CH-(CH_2_)_2_). ^13^C NMR (101 MHz, MeOD) δ 120.75 (1C, C1), 120.29 (1C, C5′), 119.40 (1C, C2), 118.57 (1C, C4′), 117.80 (1C, C6′), 85.21 (1C, C5), 63.71 (1C, C12”), 62.50 (1C, C9), 58.90 (1C, N–CH_2_–CH-(CH_2_)_2_), 43.94 (3C, C3″, C6″, C9”), 41.57 (1C, C16), 34.65 (1C, C8), 30.28 (1C, C15), 22.49 (1C, C10), 6.83 (1C, N–CH_2_–CH-(CH_2_)_2_), 6.21 (1C, N–CH_2_–CH-(CH_2_)_2_), 3.36 (1C, N–CH_2_–CH-(CH_2_)_2_). MS: C_34_H_39_N_7_O_7_ calc. 657.29. ESI (*m*/*z*): 658.30 [M + H]^+^, 329.85 [M + 2H]^2+^.

**6-Cy5**-1-(1-(((4*bS*,8*R*,8*aS*,14*bR*)-7-(cyclopropylmethyl)-1,8a-dihydroxy-5,6,7,8,8a,9,14,14b-octahydro-4,8-methanobenzofuro[2,3-*a*]pyrido[4,3-*b*]carbazol-13-yl)amino)-1,4,7,10,13-pentaoxo-3,6,9,12-tetraazaoctadecan-18-yl)-3,3-dimethyl-2-((1*E*,3*E*)-5-((*E*)-1,3,3-trimethyl-5-sulfoindolin-2-ylidene)penta-1,3-dien-1-yl)-3H-indol-1-ium-5-sulfonate.

Cy5 NHS ester Na salt (5.5 mg, 1.05 equiv.) was added in a solution of compound **5** (5 mg, 1 equiv.) in 0.8 mL of dry DMF and two droplets of DIPEA under stirring and light exclusion. The reaction mixture was left to stir for 2 days under an Ar atmosphere and darkness at RT. LCMS control (647 and 254 nm detection) showed that the reaction was concluded. DMF was carefully removed using N_2_ gas with the help of a small amount of methanol (azeotrope). The residue was dissolved in MeOH/H_2_O and purified via prep HPLC (Table S2). 1.2 mg of 99% pure product was obtained. Yield 12%. MS: C_66_H_75_N_9_O_14_S_2_ calc. 1281.49. ESI (*m*/*z*): 1283.65 [M + 1 + H]^+^, 643.25 [M + 1 + 2H]^2+^.^#^ HR-ESI-TOF-MS [M + 2H]^2+^ calcd. for C_66_H_77_N_9_O_14_S_2_: 641.7510, found: 641.7508.

**6-Cy3**-1-(1-(((4*bS*,8*R*,8*aS*,14*bR*)-7-(cyclopropylmethyl)-1,8a-dihydroxy-5,6,7,8,8a,9,14,14b-octahydro-4,8-methanobenzofuro[2,3-*a*]pyrido[4,3-*b*]carbazol-13-yl)amino)-1,4,7,10,13-pentaoxo-3,6,9,12-tetraazaoctadecan-18-yl)-3,3-dimethyl-2-((*E*)-3-((*E*)-1,3,3-trimethyl-5-sulfoindolin-2-ylidene)prop-1-en-1-yl)-3H-indol-1-ium-5-sulfonate.

Cy3 NHS ester (4.7 mg, 1.05 equiv.) was added to a solution of compound **5** (4.5 mg, 1 equiv.) in 0.8 mL of dry DMF and 2 droplets of DIPEA under stirring and light exclusion. The reaction mixture was left to stir for 2 days under an Ar atmosphere and darkness at room temperature. The LC–MS control (550 and 254 nm detection) showed that the reaction was concluded. DMF was carefully removed using N_2_ gas, with the help of a small amount of methanol (azeotrope). The residue was dissolved in MeOH/H_2_O and purified via prep HPLC (Table S2). 1.3 mg of 96% pure product was obtained. Yield 15%. MS: C_64_H_73_N_9_O_14_S_2_ calc. 1255.47. ESI (*m*/*z*): 1257.20 [M + 1 + H]^+^, 629.05 [M + 1 + 2H]^2+^.^#^ HR-ESI-TOF-MS [M + 2H]^2+^ calcd. for C_64_H_75_N_9_O_14_S_2_: 628.7432, found: 628.7432.

### Pharmacology Assays

The solid fluorescent ligands were dissolved in DMSO stock solutions (500, 800 μM) which were employed accordingly for dilutions with the medium used in each pharmacological and microscopy assay.

### Radioligand Binding Studies

The determination of binding affinities toward the human δOR, κOR, and μOR was performed, as previously described.^97,98^ Briefly, HEK293T cells were for membrane preparation, after transient transfection with the cDNAs for δOR, κOR (both cDNAs obtained from the cDNA resource center, www.cdna.org), and μOR, respectively (a generous gift from the Ernest Gallo Clinic and Research Center, UCSF, CA). Receptor densities (*B*_max_ value) and specific binding affinities (*K*_d_ value) for the radioligand [^3^H]diprenorphine (specific activity 31 Ci/mmol, PerkinElmer, Rodgau, Germany) were determined to be 1,900 ± 530 fmol/mg protein, 0.27 ± 0.06 nM for δOR, 4,400 ± 3,000 fmol/mg protein, 0.12 ± 0.02 nM for κOR, and 1900 ± 490 fmol/mg protein, 0.090 ± 0.01 nM for μOR, respectively. The protocol for competition binding experiments consisted in incubating membranes in binding buffer (50 mM Tris, 5 mM MgCl_2_, 0.1 mM EDTA, 5 μg/mL bacitracin and 5 μg/mL soybean trypsin inhibitor at pH 7.4) at a final protein concentration of 2–14 μg/well, together with the radioligand (final concentration 0.2–0.3 nM for δOR, κOR, and μOR) and varying concentrations of the competing ligands for 60 min at 37 °C. Nonspecific binding was determined in the presence of naloxone at a final concentration of 10 μM. The protein concentration was established using the method of Lowry.^99^ The resulting competition curves were analyzed by nonlinear regression using the algorithms implemented in PRISM 6.0 (GraphPad Software, San Diego, CA) to provide an IC_50_ value, which was subsequently transformed into the *K*_*i*_ value employing the equation of Cheng and Prusoff.^100^*K*_i_ values are the means of two to five single experiments all done in triplicates and are presented in [nM ± SD].

### Accumulation of Inositol Mono Phosphate (IP) as Functional Assay for G-Protein-Mediated Signaling

The IP-One HTRF assay (Cisbio, Codolet, France) was applied to determine and measure the activation of δOR, in accordance with the manufacturer’s protocol and as described previously.^87^ Briefly, after HEK293T cells were grown to reach a confluence of approximately 70%, they were transiently cotransfected with the cDNA of the human δOR (cDNA Resource Center, Bloomsburg, PA) and of the hybrid G-protein Gα_qi_ (Gα_q_ protein with the last five amino acids at the C-terminus replaced by the corresponding sequence of Gα_i_; gift from The J. David Gladstone Institutes, San Francisco, CA)^86^ using the Mirus TransIT-293 transfection reagent (Peqlab, Erlangen, Germany). After 1 day, cells were detached from the culture dish with Versene (Life Technologies, Darmstadt, Germany), seeded into black 384-well plates (10,000 cells/well) (Greiner Bio-One, Frickenhausen, Germany), and maintained for 24 h at 37 °C. The determination of agonist properties was performed by incubating the test compounds (final range of concentration from 1 pM up to 10 μM) in duplicates for 90 min at 37 °C. For the determination of antagonist properties, cells were pre-incubated with the test compound for 30 min, followed by adding 30 nM of leu-enkephalin (EC_80_ concentration) and continuing incubation for 90 min. Accumulation of the second messenger was stopped by adding detection reagents (IP1-d2 conjugate and Anti-IP1cryptate TB conjugate) and monitoring time-resolved FRET with a Clariostar plate reader (BMG Labtec, Ortenberg, Germany). FRET ratios were calculated as the ratio of emission intensity of the FRET acceptor (665/10 nm) divided by the FRET donor intensity (620/10 nm). Raw FRET ratios were normalized to buffer conditions (0%) and the maximum effect of leu-enkephalin (100%), and the obtained responses were analyzed using the equation for sigmoid concentration–response curves (four-parameter) implemented in GraphPad Prism 6.0 (GraphPad Software, La Jolla, USA) to derive the maximum efficacy (*E*_max_, relative to leu-enkephalin) and ligand potency (EC_50_). For antagonist studies, the maximum effect at 30 nM leu-enkephalin was normalization to 100%. For each compound, eight to nine independent experiments were performed with each concentration in duplicate (Figure S1, Table S3).

### Recruitment of β-Arrestin-2

Arrestin-2 recruitment was measured using the PathHunter assay (DiscoverX, Birmingham, U.K.), in accordance with the manufacturer’s protocol and as described previously.^87,88^ Briefly, a HEK293T cell culture stably expressing the enzyme acceptor (EA) tagged β-arrestin-2 fusion protein was transiently transfected with the ProLink tagged δOR-PK2 construct, using the Mirus TransIT-293 transfection reagent. After 24 h, cells from the above culture were transferred into white clear bottom 384-well plates (5000 cells/well) (Greiner Bio-One) and maintained for an additional 24 h under conditions 37 °C, 5% CO_2_. To determine receptor-stimulated β-arrestin-2 recruitment, solutions of the tested compounds were added to the wells to obtain a final concentration in the range of 10 pM to 10 μM. Incubation was continued for 90 min at 37 °C. Antagonist properties were measured by preincubation with the test compound for 30 min, followed by adding 100 nM of leu-enkephalin (EC_80_ concentration) and continuing incubation for 90 min. Stimulation was stopped by the addition of a detection mix and further incubation for 60 min at room temperature. Chemiluminescence was determined using a Clariostar plate reader. Data analysis was done as described for the IP_1_ accumulation assay. For each compound, five to nine independent experiments were performed with each concentration applied in duplicate (Figure S2, Table S3).

### HTRF Assay

#### Materials and Methods for Affinity and Selectivity Study via HTRF

Dulbecco’s Modified Eagle’s Medium (DMEM) and fetal bovine serum (FBS) were obtained from Life Technologies (Grand Island, NY, USA). SNAP-opioid receptor plasmids and BG-Lumi4-Tb were commercialized by CisBio bioassays and provided by Dr. S. Granier.

#### Cell Culture and Transfection

HEK293T cells (from ATCC) were grown in DMEM supplemented with 10% FBS (without antibiotics) at 37 °C and 5% CO_2_. Transient transfection was performed using electroporation in a volume of 200 μL with 1 μg of SNAP-opioid plasmids and 10 million HEK293T cells in electroporation buffer (50 mM K_2_HPO_4_, 20 mM CH_3_COOK, and 20 mM KOH, pH 7.4). After electroporation (250 V, 500 μF, Bio–Rad Gene Pulser electroporator; Bio-Rad Laboratories, Hercules, CA), cells were resuspended in 10 mL of DMEM supplemented with 10% fetal bovine serum and seeded for 24 h in a white Greiner Bio-One 96-well plate (pretreated with Poly-l-Ornithine 1X) at a density of 100,000 cells per well.

#### SNAP-tag Labeling

Twenty-four hours after transfection, SNAP-receptors were labeled with 100 nM of BG-Tb (benzylguanine-terbium cryptate) for 1 h at 37 °C in Tag-Lite buffer (commercialized by Reavvity). After four washing steps with PBS, the fluorescence signal from BG-Lumi4-Tb was measured on a SPARK20 M plate reader (TECAN) with an excitation at 337 nm and an emission at 620 nm.

#### Fluorescent Ligand-Binding Assay

HEK293T cells expressing SNAP-opioid receptors and labeled with Lumi4-Tb were incubated with increasing concentrations of fluorescent ligands for 1 h at room temperature (from 0.1 to 100 nM) ± an excess of naloxone (100 μM). The sample volume was 100 μL/well.

#### Signal Detection

HTRF signal detection was performed on a SPARK20 M instrument (TECAN). The signal was collected both at 665 and 620 nm. HTRF ratios were obtained by dividing the acceptor signal at 665 nm by the donor signal at 620 nm and multiplying the obtained ratios by 10,000. The integration time of the HTRF was 60–400 μs. Data were then analyzed using GraphPad Prism (GraphPad Software, Inc., San Diego, CA). *K*_d_ values of the fluorescent ligands were obtained from saturation curves of specific binding.

### Assays on the TIRF Microscope

#### Cell Culture

For the microscopy experiments, Chinese hamster ovary (CHO) K1 cells (Leibniz-Institute DSMZ-German Collection of Microorganisms and Cell Culture) were kept in 10 cm Petri dishes with phenol red-free DMEM/F12 medium supplemented with 10% fetal bovine serum (FBS), 100 U/mL penicillin, and 100 μg/mL streptomycin at 37 °C and 5% CO_2_.

#### Transfection

The day before transfection, CHO cells were seeded at a density of 1.8 × 10^5^ cells per well on ultraclean 24 mm glass coverslips in six-well culture plates. Transfection was performed with Lipofectamine2000 (Thermo Fisher Scientific) according to the manufacturer’s protocol. For each well, 6 μL of Lipofectamine2000 and 2 μg of wildtype δOR (a generous gift from the Kobilka Lab, Stanford University, CA) were used.

#### Fluorescent Ligand Binding Experiments

After 24 h of transfection, fluorescent ligand binding experiments were performed. Therefore, each of the aforementioned coverslip culture samples was incubated with the respective concentration of fluorescent ligands dissolved in a medium for 20 min at 37 °C. Before imaging, the sample was once rapidly washed with 1 mL of medium followed immediately by mounting to the microscopic chamber filled with 400 μL of the medium. Imaging was performed on a customized Nikon Eclipse Ti TIRF microscope using a 60x oil-immersion objective (CFI Apochromat TIRF 60× oil NA 1.49). Both the sample and objective were kept at 37 °C with a water-cooling system.

Cells were searched and imaged using a 561 nm diode laser for compound **6-Cy3** and a 638 nm diode laser for compound **6-Cy5** (both lasers from Coherent). At least 50 cells per condition from three independent experiments were analyzed using FIJI. The fluorescent intensity values were corrected for background fluorescence and normalized to the values obtained for the highest concentration. Fitting was performed using a one-site ligand binding model with a Hill slope of 1 in GraphPad Prism6.

#### Dissociation Kinetics Experiments

Dissociation kinetic experiments were performed 24 h after transfection. Each sample was incubated with a saturating concentration (1 μM) of either compound **6-Cy3** or **6-Cy5** for 20 min at 37 °C. Afterward, the coverslips were mounted in a microscopic chamber filled with 400 μL of medium and washed once rapidly while being mounted on the microscope. Imaging was performed on the same TIRF microscope described above, and images were acquired every minute using the 561 nm for compound **6-Cy3** and 638 nm laser for compound **6-Cy5**, respectively.

The acquired images were analyzed using FIJI. To obtain the background-corrected average fluorescent intensity of each cell in each image of the time series, a region of interest (ROI) was manually defined for each cell. The intensities were then normalized to the initial intensity at the beginning of the wash, and data was fitted to a one-phase exponential decay in Prism 6. Control experiments for photobleaching were performed using the same number of frames and laser intensities, showing that the effect of bleaching is negligible.

### Single Molecule Microscopy

#### Experiment

CHO-K1 cells (ATCC) were cultured in phenol red-free DMEM/F12, supplemented with 10% FBS at 37 °C with 5% CO_2_. Cells were seeded onto 25 mm clean glass coverslips at a density of 3 × 10^5^ per well. On the following day, cells were transfected with δOR and SNAP-CD86 (as the control) constructs, using Lipofectamine 2000, in accordance with the recommendations of the manufacturer. Four hours after the transfection, cells were labeled with 100 nM of **6-Cy3** and **6-Cy5** for 20 min, while SNAP-CD86 transfected cells were labeled with 1 μM SNAP-Alexa 647 (New England Biolabs, UK) in a complete culture medium. After 3 × 5 min of wash, single-molecule microscopy experiments were performed using total internal reflection fluorescence (TIRF) illumination on a custom system (assembled by CAIRN Research) based on an Eclipse Ti2 microscope (Nikon) equipped with a 100x oil immersion objective (SR HP APO TIRF NA 1.49, Nikon), 405, 488, 561, and 637 nm diode lasers (Coherent, Obis), an iLas2 TIRF illuminator (Gataca Systems), quadruple band excitation and dichroic filters, a quadruple beam splitter, 1.5x tube lens, four EMCCD cameras (iXon Ultra 897, Andor), hardware focus stabilization, and a temperature-controlled enclosure. The sample and objective were maintained at 37 °C throughout the experiments. Coverslips were mounted in a microscopy chamber filled with HBSS supplemented with 10 mM HEPES, at pH 7.5. Dual-color single-molecule image sequences were acquired simultaneously on synchronized EMCCDs at a rate of one image every 30 ms. Only individual cells with comparable expression levels on both channels were selected for single-molecule analyses. The recording settings were 400 frames for each cell (1 movie is 1 cell) and one frame is 30 ms, which is 12 s for each movie. For single-color SMM 54 movies were recorded. For the dual-color SMM, 24 δOR-CD86 control movies and 54 δOR-δOR films were recorded. In total, that is, 132 movies sum to 1584 s. The videos were analyzed as image sequences with an automated particle detection software (utrack) in the MATLAB environment. Further investigation followed using custom algorithms, as previously described.^90,91^

#### Analysis

The time-averaged mean squared displacement (TA-MSD)^92^ of individual particle trajectories from TIRF image sequences was computed to analyze the motion of receptors, as previously described.^91^ The TA-MSD data were fitted with the following equation to calculate the diffusion coefficient (*D*):
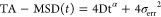
where *t* indicates time, α is the anomalous diffusion exponent, and σ_err_ is a constant offset for the localization error. Only trajectories lasting at least 100 frames were analyzed (6100–11,000 trajectories in each group). A classification of the trajectories according to the diffusion parameters D and α followed. Particles with *D* < 0.01 μm^2^s^–α^ were classified as immobile. Particles classified under normal diffusion had *D* ≥ 0.01 μm^2^s^–α^ and 0.75 ≤ α ≤ 1.25. Particles adhering to the sub- and superdiffusion classifications had *D* ≥ 0.01 μm^2^s^–α^ and α < 0.75 or α > 1.25, respectively.

To analyze dimer formation, trajectory segments were first linked in order to obtain continuous trajectories that are not interrupted by merging and splitting events. Afterward, for each particle in the Cy5 channel at frame f, all particles in the Cy3 channel falling within a defined search radius (150 nm) were identified as colocalizing. If a colocalization was also present at frame *f* + 1, then the colocalization was extended. The process was iterated until the last frame of the image sequence. These data were used to build a matrix containing information for each colocalization (involved particles as well as the start and end frames). The observed colocalization time corresponds to the average duration of true interactions plus the average duration of random colocalizations. Thus, the distribution of the observed colocalization times can be seen as a convolution of the distribution of true interaction times and random colocalization times. The expected distribution for random colocalizations was measured using the δOR (labeled with **6-Cy3**) and a noninteracting membrane protein (CD86, labeled with SNAP Alexa 647). To obtain the true colocalization time, deconvolution with the Lucy–Richardson algorithm was performed.^91^

## Supporting Information Available

Details about the purification and characterization of the ligands (liquid chromatography elution methods, LC–MS reports of target compounds, ^1^H NMR spectra), fluorescence binding curves, and intrinsic activity assay (PDF)
Molecular formula strings (CSV)

## Notes

This work is part of the doctoral thesis of Antonios Drakopoulos^101^.

## Abbreviations

BRET: bioluminescence resonance energy transfer
Cbz: benzyloxycarbonyl
CHO: Chinese hamster ovary
CNS: central nervous system
Cy3: cyanine dye 3
Cy5: cyanine dye 5
DIPEA: *N*,*N*-diisopropylethylamine
DMEM: Dulbecco’s Modified Eagle’s Medium
DMF: dimethylformamide
δOR: delta opioid receptor
EDCI: 1-ethyl-3-(3-dimethylaminopropyl)carbodiimide
EMCCD: electron-multiplying charge-coupled device
FBS: fetal bovine serum
Fmoc: 9-fluorenylmethoxycarbonyl
FRET: Förster resonance energy transfer
GFP: green fluorescent protein
GPCR: G protein-coupled receptor
HBSS: Hank’s balanced salt solution
HEK: human embryonic kidney
HEPES: 4-(2-hydroxyethyl)-1-piperazineethanesulfonic acid
HOBt: hydroxybenzotriazole
HPLC: high-performance liquid chromatography
HTRF: Homogeneous Time-Resolved FRET
κOR: kappa opioid receptor
LCMS: liquid chromatography–mass spectrometry
μOR: mu opioid receptor
NHS: *N*-hydroxysuccinimide
NOR: nociception receptor
NTI: naltrindole
OR: opioid receptor
RP: reverse-phased
SAR: structure–activity relationships
SMM: single-molecule microscopy
TA-MSD: time-averaged mean squared displacement
TFA: trifluoroacetic acid
TIRF: total internal fluorescence
TLC: thin layer chromatography
