# Supplementary material for: Design, Synthesis, and Characterization of New δ Opioid Receptor-Selective Fluorescent Probes and Applications in Single-Molecule Microscopy of Wild-Type Receptors
Source: J Med Chem. 2024 Jul 24;67(15):12618–31. doi: 10.1021/acs.jmedchem.4c00627 (PMC11386433; doi:10.1021/acs.jmedchem.4c00627)
Supplement: Supplementary file 1 — jm4c00627_si_001.pdf [file jm4c00627_si_001.pdf]

Supporting Information

Design, synthesis and characterization of new δ opioid receptor-selective fluorescent probes and applications in Single-Molecule Microscopy of wild-type receptors

Antonios Drakopoulos[1], Zsombor Koszegi[2,3], Kerstin Seier[4], Harald Hübner[5], Damien Maurel[6], Rémy Sounier[6], Sébastien Granier[6], Peter Gmeiner[5], Davide Calebiro[2,3], Michael Decker*[,1]

[1]Pharmazeutische und Medizinische Chemie, Institut für Pharmazie und Lebensmittelchemie, Julius-Maximilians-Universität Würzburg, Am Hubland, 97074, Würzburg, Germany
[2]Institute of Metabolism and Systems Research, University of Birmingham, B15 2TT, Birmingham, United Kingdom
[3] Centre of Membrane Proteins and Receptors, Universities of Birmingham and Nottingham, B15 2TT, Birmingham, United Kingdom
[4]Institute of Pharmacology and Toxicology, Julius Maximilian University of Würzburg, Versbacher Strasse 9, 97078, Würzburg, Germany
[5]Chair of Pharmaceutical Chemistry, Department of Chemistry and Pharmacy, Friedrich-Alexander University of Erlangen-Nürnberg, 91058 Erlangen, Germany
[6]Institut de Génomique Fonctionnelle, Université de Montpellier, CNRS, INSERM, 34094 Montpellier Cedex 5, France.

Corresponding author

*Michael Decker: michael.decker@uni-wuerzburg.de

Contents

1. S2: Semi-prep HPLC method for compounds **5**, **6-Cy3** and **6-Cy5**
2. S3: IP- and β-arrestin-2 functional assays
3. S5: Microscopy part: TIRF imagine and fluorescent ligand binding experiment
4. S7: NMR spectra
5. S9: Semi-preparative HPLC data

Experimental part

Chemistry part

Materials and methods

Liquid chromatography -/ mass spectrometry - data

The purification of the precursor **5** and target compounds **6-Cy3**, **6-Cy5** was performed via semi-preparative HPLC on the Shimadzu system (cf. manuscript) using a puri a Synergi 4u Fusion-RP 80A (250×10.0 mm) column as stationary phase and the flow rate was 2.5-3 mL/min. The gradient system used for compound **5** is described below:

Table S1: Semi-prep HPLC method for compound 5

Time (min)	[A] % (methanol 0.1% HCOOH)	[B] % (water 0.1% HCOOH)
0	10	90
0-15	10-60	90-40
15-25	60	40
25-26	60-90	40-10
26-30	90	10

Compounds **6-Cy3**, **6-Cy5** were purified via semi-prep HPLC with the method:

Table S2: Semi-prep HPLC method for compounds 6-Cy3 and 6-Cy5

Time (min)	[A] % (methanol 0.1% HCOOH)	[B] % (water 0.1% HCOOH)
0	5	95
0-4	5-25	95-75
4-10	25	75
10-13	60-90	40-10
13-16	90	10
16-19	90-70	10-30
19-22	70-50	30-50
22-25	50-20	50-80
25-27	20-5	80-95
27-30	5	95

Pharmacology assays

Accumulation of inositol mono phosphate (IP) as functional assay for G-protein mediated signaling.

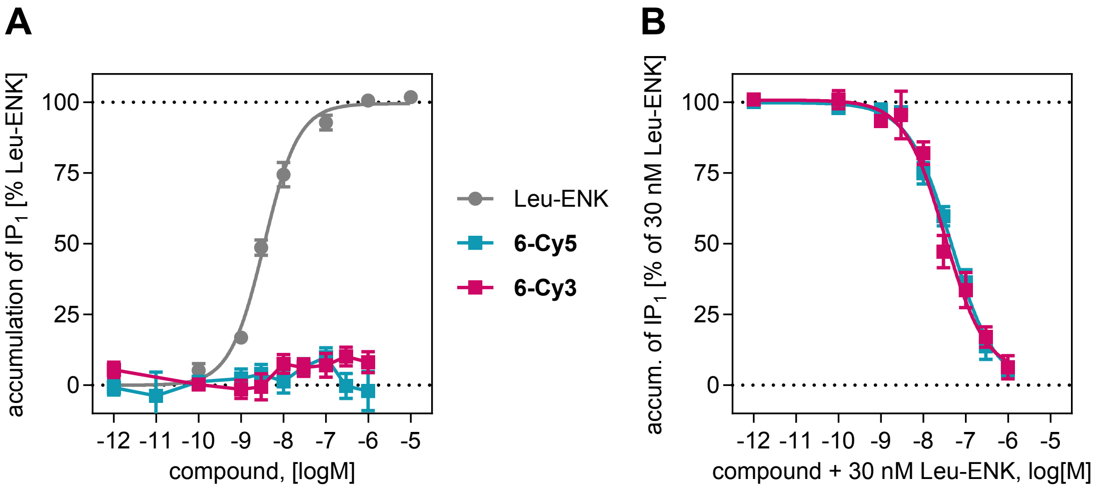

Figure S1: Accumulation of IP1 as a functional assay for δOR activation. A) Agonist properties of 6-Cy3 and 6-Cy5 relative to the full effect of the reference compound leu-enkephalin. B) Inhibitory effect of 6-Cy3 and 6-Cy5 against the agonist effect of 30 nM of leu-enkephalin. Curves represent mean values ± SEM of 8 (A) or 9 (B) independent experiments each done in duplicates.

Recruitment of β-arrestin-2.

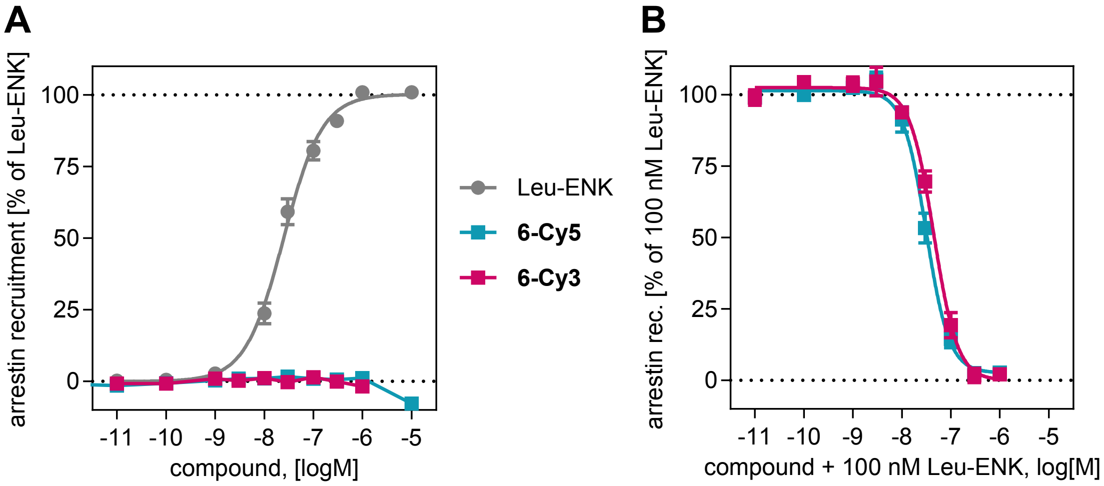

Figure S2: β-Arrestin-2 recruitment as a functional assay for δOR activation. A) Agonist properties of 6-Cy3 and 6-Cy5 relative to the full effect of the reference compound leu-enkephalin. B) Inhibitory effect of 6-Cy3 and 6-Cy5 against the agonist effect of 100 nM of leu-enkephalin. Curves represent mean values ± SEM of 5 (6-Cy3 in A), 8 (Leu-ENK in A), or 9 (6-Cy3 in B, 6-Cy5 in A, B) independent experiments each done in duplicates.

Table S3: Functional properties of the fluorescence ligands 6-Cy3 and 6-Cy5 in comparison to the reference agonist leu-enkephalin at the δOR determined with an IP_1 accumulation assay monitoring G-protein signaling and with a β-arrestin-2 recruitment assay.

compd.	agonist properties			antagonist properties[a]	
	EC_{50} [nM ± S.E.M.][b]	E_{max} [% ± S.E.M.][c]	n[d]	IC_{50} [nM ± S.E.M.][e]	n[d]
	IP_1 accumulation				
leu-enkephalin	3.9 ± 0.69	100	8	--	--
**6-Cy3**	n.a.	<10	8	38 ± 6.2	9
**6-Cy5**	n.a.	<5	8	54 ± 9.0	9
	β-arrestin-2 recruitment				
leu-enkephalin	27 ± 4.2	100	8	--	--
**6-Cy3**	n.a.	<5	5	47 ± 4.6	9
**6-Cy5**	n.a.	<5	9	33 ± 3.5	9

[a] Antagonist properties were determined by measuring the inhibition of the agonist effect induced by 30 nM (for IP_1 assay) or 100 nM (for arrestin recruitment) of leu-enkephalin (EC_{80} concentration). [b] Potency for δOR activation in nM ± S.E.M. [c] Maximum efficacy in % ± S.E.M. relative to the full effect of leu-enkephalin. [d] Number of experiments each done in duplicates. [e] Potency of the inhibitory effect of the test compound against the agonist effect of leu-enkephalin in nM ± S.E.M. n.a.: Could not be analyzed because of the lack of a sigmoidal dose-response curve.

Microscopy part

Assays on TIRF microscope

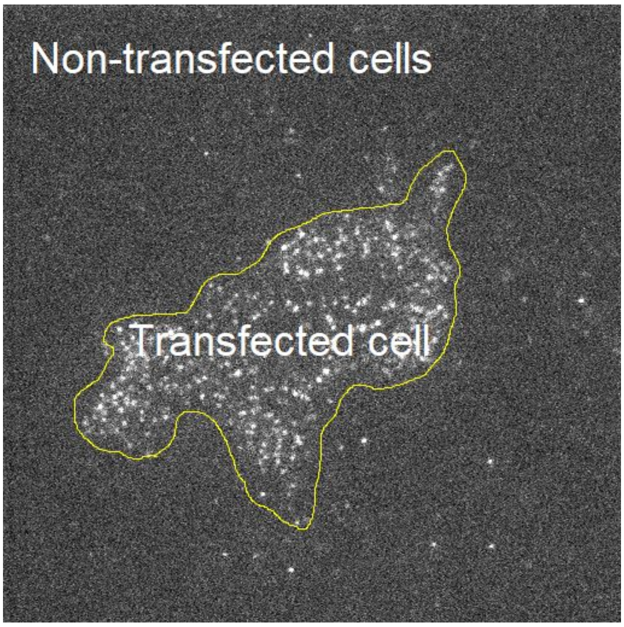

Figure S3: An exemplary TIRF microscopy image of a transfected cell surrounded by non-transfected cells on a confluent coverslip.

Fluorescent ligand binding experiments

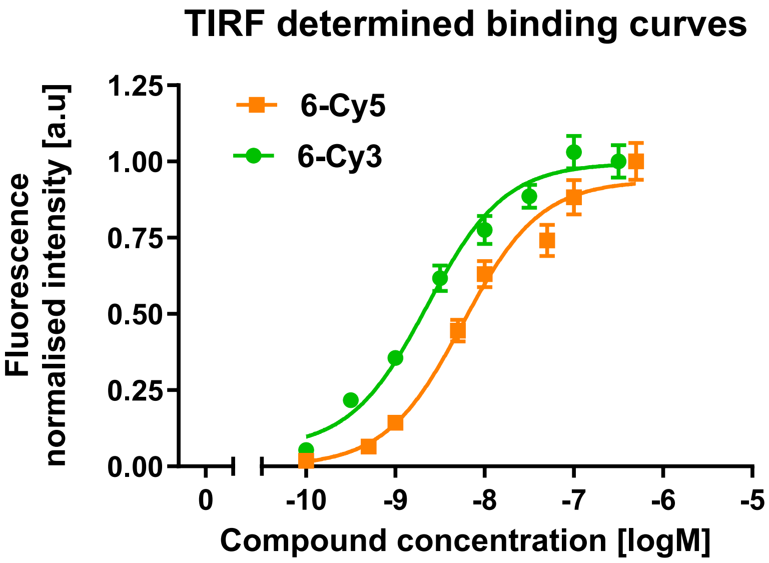

Figure S4: TIRF determined binding curves for 6-Cy5 (orange) and 6-Cy3 (green). The intensity of all concentrations was normalized to the intensity of the highest concentration. Each data point is the average of three independent experiments ± S.E.M.

Table S4: Affinity and selectivity measurements for 6-Cy5 and 6-Cy3

	$K_{i/d}$ (nM) δOR	$K_{i/d}$ (nM) μOR	$K_{i/d}$ (nM) κOR	μ/δ	κ/δ
**6-Cy5 (HTRF)**	1.8 ± 0.8	215 ± 262[a]	226 ± 131[a]	120[a]	126[a]
**6-Cy5 (radiolig.)**	1.2 ± 0.75	100 ± 16	78 ± 28	83	65
**6-Cy5 (TIRF)**	5.7 ± 2.4	n.d.	n.d.	n.d.	n.d.
**6-Cy3 (radiolig.)**	1.7 ± 0.51	370 ± 35	330 ± 85	220	190
**6-Cy3 (TIRF)**	2.3 ± 0.9	n.d.	n.d	n.d.	n.d.

a: Values of high uncertainty. Ligand saturation level cannot be reached for these receptors within the concentration range of the specific assay settings.

Appendix

NMR spectra

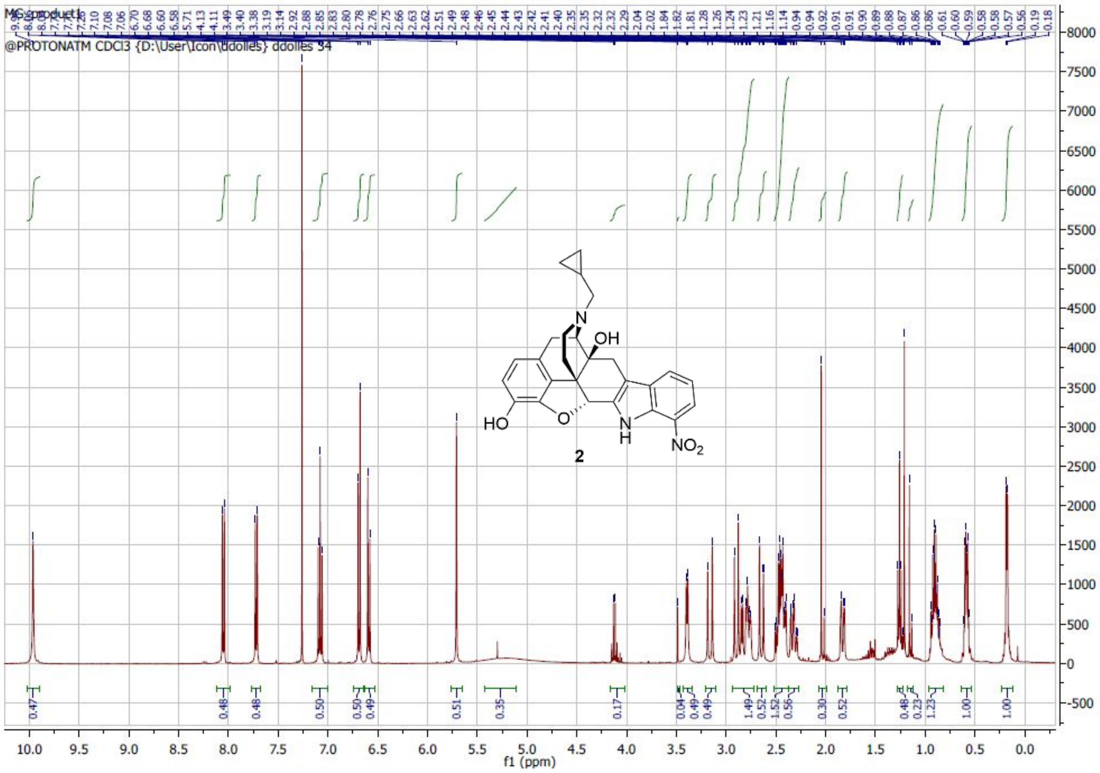

2 - 7’-nitronaltrindole

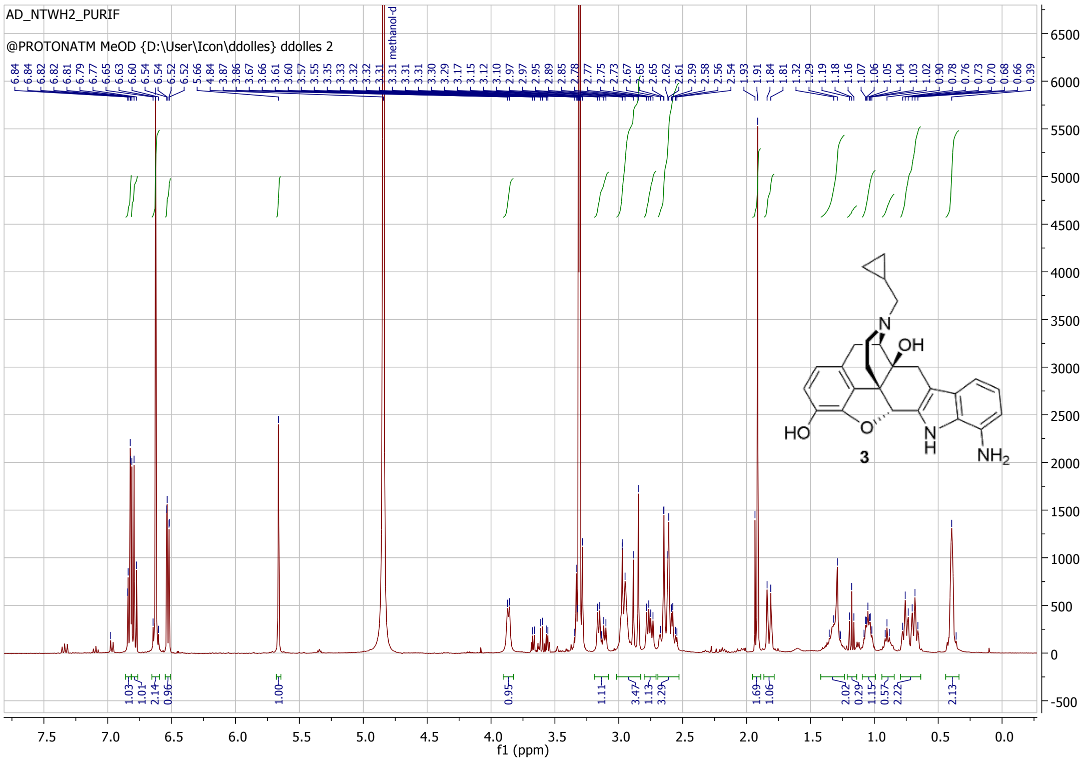

3 - 7’-aminonaltrindole

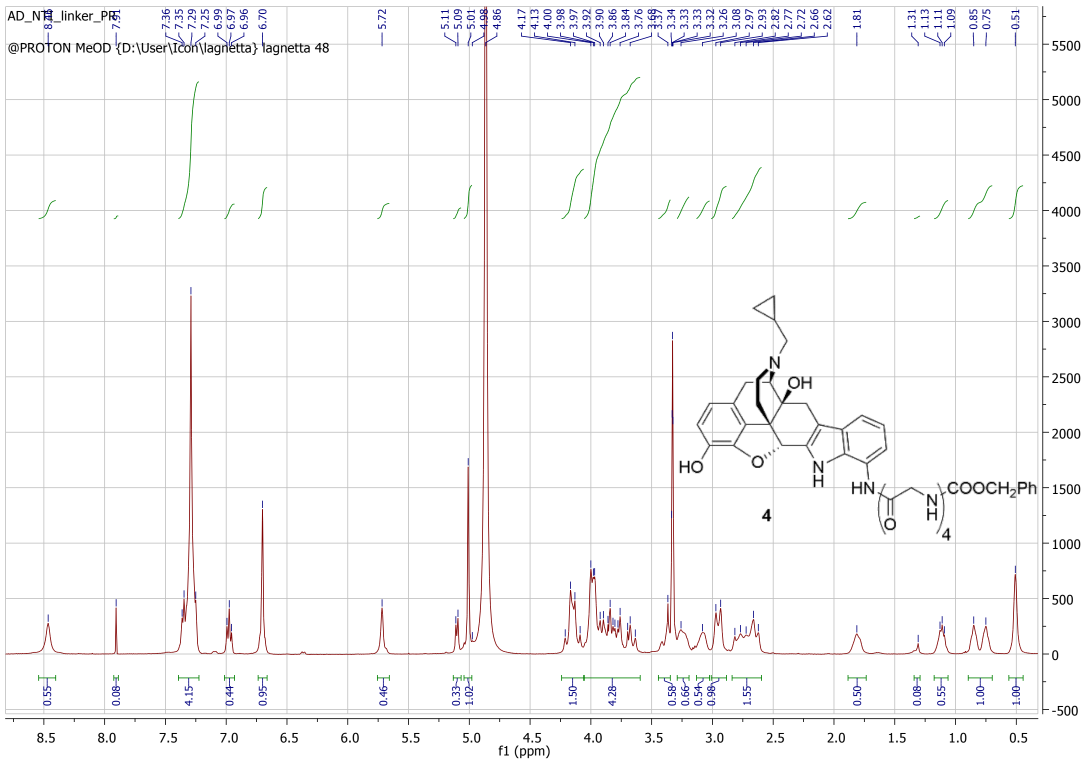

4 - 7’-(*N*-Cbz-tetraglycyl)-amidonaltrindole

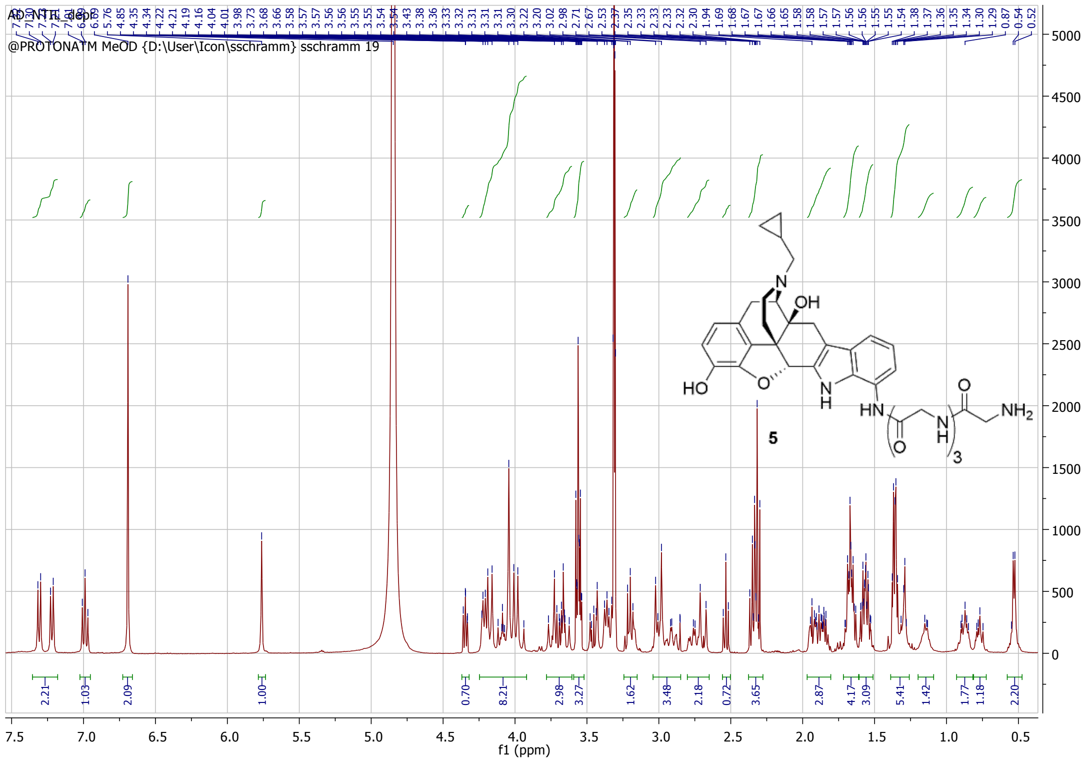

5 – 7’-tetraglycylamidonaltrindole

LCMS data

LCMS elution gradient methods

Table S5: LCMS method 05-90

Time (min)	[A] % (methanol 0.1% HCOOH)	[B] % (water 0.1% HCOOH)
0	5	95
0-8	5-90	95-10
8-13	90	10
13-14	90-5	10-95
14-18	5	95

Table S6: LCMS method 05-80

Time (min)	[A] % (methanol 0.1% HCOOH)	[B] % (water 0.1% HCOOH)
0	5	95
0-10	5-80	95-20
10-15.5	80	20
15.5-16.5	80-5	20-95
16.5-18	5	95

Table S7: LCMS method 0-60

Time (min)	[A] % (methanol 0.1% HCOOH)	[B] % (water 0.1% HCOOH)
0	0	100
0-10	0-60	100-40
10-14	60	40
14-15	60-0	40-100
15-18	0	100

Table S8: LCMS method 05-50

Time (min)	[A] % (methanol 0.1% HCOOH)	[B] % (water 0.1% HCOOH)
0	5	95
0-10	5-50	95-50
10-15.5	50	50
15.5-16.5	50-5	50-95
16.5-18	5	95

LCMS reports

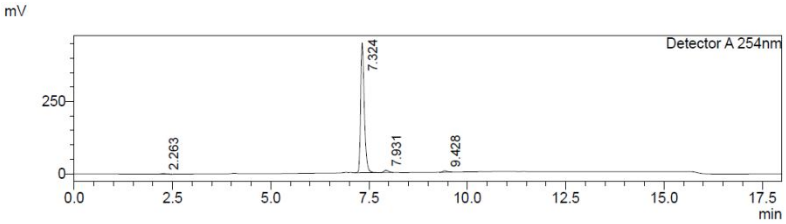

Chromatogram of 7’-nitronaltrindole **2** - method 05-90

Detector A 254nm

Peak#	Ret. Time	Area	Height	Area%
1	2.263	17157	1805	0.571
2	7.324	2897586	446863	96.510
3	7.931	55843	7263	1.860
4	9.428	31785	4709	1.059
Total		3002371	460641	100.000

Chromatogram peak table of of 7’-nitronaltrindole **2** - method 05-90

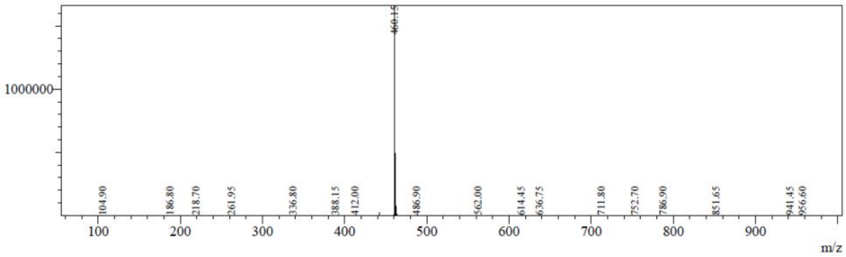

Mass sprectrum of 7’-nitronaltrindole **2** - m/z: +60-1000

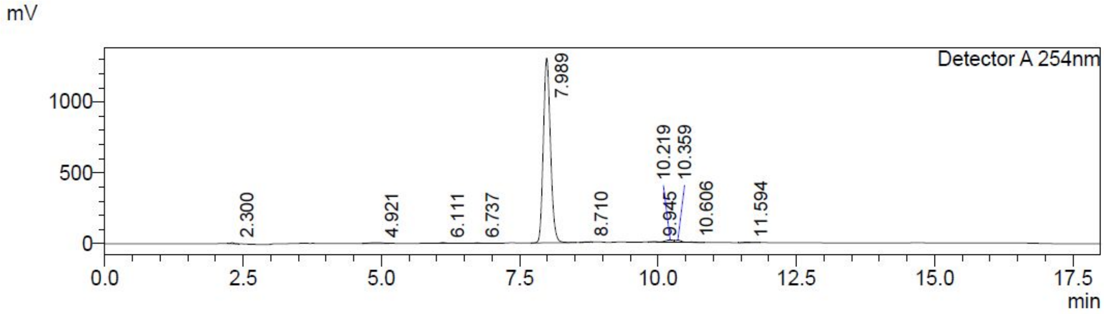

Chromatogram of 7’-aminonaltrindole **3** - method 0-60

Detector A 254nm

Peak#	Ret. Time	Area	Height	Area%
1	2.300	22275	5735	0.182
2	4.921	87684	5488	0.718
3	6.111	39123	5715	0.320
4	6.737	22339	3226	0.183
5	7.989	11692928	1303991	95.709
6	8.710	21466	3122	0.176
7	9.945	20900	3031	0.171
8	10.219	152745	14969	1.250
9	10.359	105120	14826	0.860
10	10.606	15264	2125	0.125
11	11.594	37275	3514	0.305
Total		12217119	1365743	100.000

Chromatogram peak table of of 7’-aminonaltrindole **3** - method 0-60

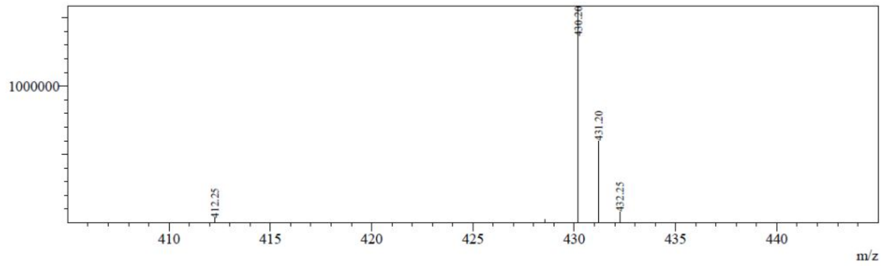

Mass sprectrum of 7’-aminonaltrindole **3** - m/z: +100-1000

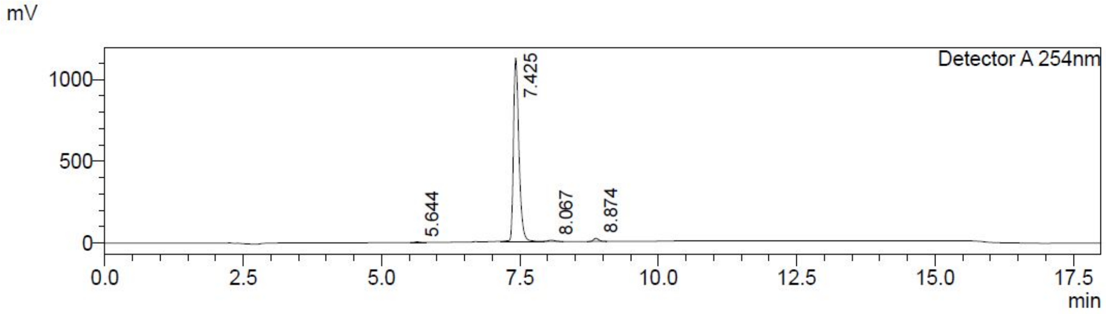

Chromatogram of 7’-(*N*-Cbz-tetraglycyl)-amidonaltrindole **4** - method 05-90

Detector A 254nm

Peak#	Ret. Time	Area	Height	Area%
1	5.644	23014	3635	0.294
2	7.425	7630336	1115308	97.391
3	8.067	58348	7462	0.745
4	8.874	123080	18725	1.571
Total		7834778	1145130	100.000

Chromatogram peak table of of 7’-(*N*-Cbz-tetraglycyl)-amidonaltrindole **4** - method 05-90

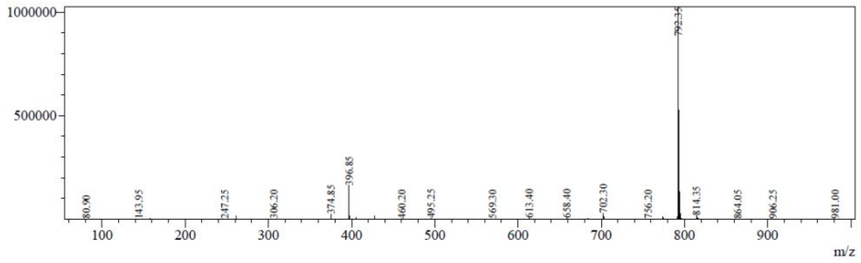

Mass sprectrum of 7’-(*N*-Cbz-tetraglycyl)-amidonaltrindole **4** - m/z: +60-1000

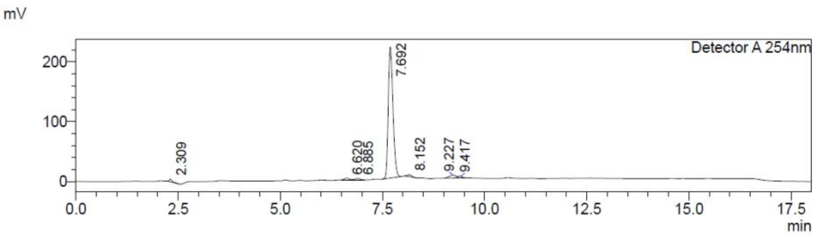

Chromatogram of 7’-tetraglycylamidonaltrindole **5** - method 0-60

Detector A 254nm				
Peak#	Ret. Time	Area	Height	Area%
1	2.309	24753	4544	1.283
2	6.620	22431	2998	1.163
3	6.885	19832	2648	1.028
4	7.692	1758912	218681	91.170
5	8.152	25086	3200	1.300
6	9.227	54744	5042	2.838
7	9.417	23508	2943	1.219
Total		1929266	240056	100.000

Chromatogram peak table of of 7’-tetraglycylamidonaltrindole **5** - method 0-60

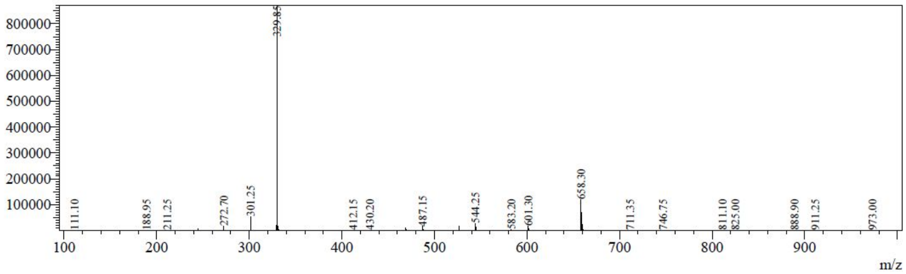

Mass sprectrum of 7’-tetraglycylamidonaltrindole **5** - m/z: +100-1000

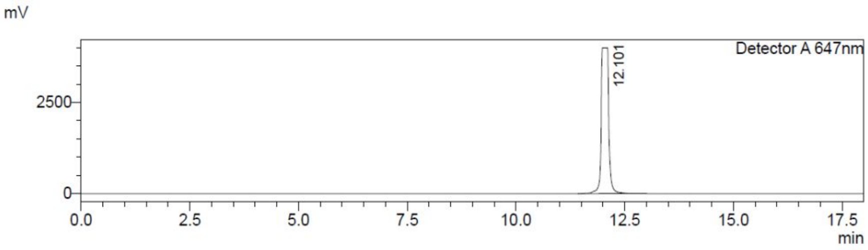

Chromatogram of **6-Cy5** - method 0-60 (647 nm)

Detector A 647nm				
Peak#	Ret. Time	Area	Height	Area%
1	12.101	47342226	3998171	100.000
Total		47342226	3998171	100.000

Chromatogram peak table of **6-Cy5** - method 0-60 (647 nm)

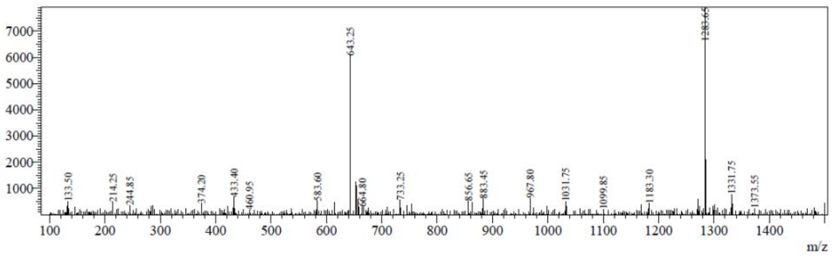

Mass sprectrum of **6-Cy5** - m/z: +100-1500

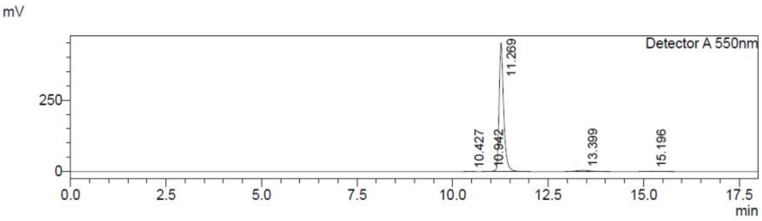

Chromatogram of **6-Cy3** - method 0-60 (550 nm)

Detector A 550nm

Peak#	Ret. Time	Area	Height	Area%
1	10.427	3196	410	0.082
2	10.942	5945	771	0.153
3	11.269	3748598	449016	96.362
4	13.399	112014	4464	2.879
5	15.196	20353	747	0.523
Total		3890106	455407	100.000

Chromatogram peak table of **6-Cy3** - method 0-60 (550 nm)

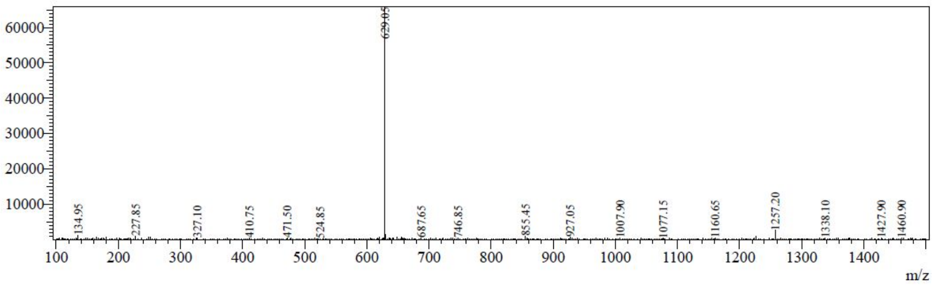

Mass sprectrum of **6-Cy3** - m/z: +100-1500
